# Time to Heal: MicroRNA Temporal Dysregulation in Diabetic Foot Ulcers

**DOI:** 10.3390/ijms27198491

**Published:** 2026-09-23

**Authors:** Lingyu Pan, Lingyun Zhao, Jinfeng Liu, You Mou, Kaili Wang, Xinyi Liu, Wei Hua

**Affiliations:** 1 Department of Dermatology and Venereology, West China Hospital, Sichuan University, Chengdu 610041, China; panlingyu@stu.scu.edu.cn (L.P.); zhaolingyun@stu.scu.edu.cn (L.Z.); 2024224020127@stu.scu.edu.cn (J.L.); 2025224025375@stu.scu.edu.cn (Y.M.); 2026224025365@stu.scu.edu.cn (K.W.); 2026224020066@stu.scu.edu.cn (X.L.); 2 Cosmetics Safety and Efficacy Evaluation Center, West China Hospital, Sichuan University, Chengdu 610041, China

**Keywords:** diabetic foot ulcer, metabolic inflammation, microRNA, wound healing, temporal regulation, angiogenesis, extracellular vesicles, chronobiology

## Abstract

Diabetic foot ulcers (DFUs) are a severe complication of diabetes characterized by delayed healing, infection susceptibility, and tissue necrosis. During normal healing, microRNA (miRNA) expression changes over time and helps regulate inflammation, tissue formation, and matrix remodeling. The same miRNA can serve opposing roles depending on cell type, timing, and wound context. Hyperglycemia, hypoxia, and oxidative stress disrupt these processes and prolong inflammation. This review examines how altered miRNA regulation may impair wound-healing transitions and vessel maturation in DFUs. We organize findings from experimental models and human samples into three interpretive patterns: delayed or insufficient induction, aberrant persistence, and temporal inversion or phase-inappropriate expression. Angiogenesis provides an example of how disrupted miRNA timing may impair vascular repair and granulation tissue formation. Circadian regulation may further influence these processes, although direct evidence linking circadian regulation, miRNA dynamics, and human DFUs remains limited. Preclinical studies of miRNA combinations, engineered vesicles, and biomaterial carriers suggest opportunities to coordinate local miRNA delivery with repair needs. The human studies reviewed here do not establish clinical efficacy of a defined miRNA mimic or inhibitor in DFUs. Further translation requires miRNA profiling over time, assessment of the wound’s biological stage, and delivery to the relevant cells during the appropriate repair window.

## 1. Introduction

Diabetic foot ulcers (DFUs) are among the most serious complications of diabetes. The lifetime risk of developing a foot ulcer in patients with diabetes has been estimated to approach one-third, and DFUs affect millions of patients worldwide, causing marked impairment in quality of life and substantial socioeconomic burden [1,2,3,4]. The pathological process of DFUs is multifaceted, involving the interaction of a hyperglycemic microenvironment, microvascular disease, neuropathy, immune dysfunction, infection risk, and recurrent tissue injury. Together, these features identify DFUs as a disorder of metabolic inflammation, in which hyperglycemia and the associated oxidative and mitochondrial stress sustain a low-grade inflammatory state that fails to resolve after injury. Clinically, DFUs are characterized by high recurrence rates, markedly increased risk of lower-extremity amputation, and increased mortality compared with diabetic patients without foot ulceration [5,6].

Standard DFU care includes pressure off-loading, debridement, infection control, revascularization when indicated, and appropriate dressings, as recommended by current IWGDF and American Diabetes Association guidance [7,8,9]. These measures address the main clinical barriers to healing. To understand persistent healing failure, it is also important to consider how repair processes are coordinated over time and across wound regions.

Recent work has examined wound repair from this temporal and spatial perspective. Agramunt et al. proposed “spatiotemporal clocks” as a framework for coordinating phase transitions across the epidermis, dermis, fascia, wound edge, and wound center [10]. A human single-cell multi-omics roadmap subsequently showed region- and phase-specific changes in wound-margin architecture, keratinocyte migration, macrophage-fibroblast interactions, and re-epithelialization [11]. In DFUs, Theocharidis et al. identified healing-associated fibroblast populations, distinct macrophage states, and spatial differences among wound bed, wound edge, and unwounded skin [12]. A 2026 secondary analysis integrating public bulk and single-cell transcriptomic datasets further reported collagen-centric signaling and altered monocyte/macrophage–endothelial communication in non-healing DFUs [13]. These studies do not establish miRNA trajectories, but they show that chronicity involves maladaptive cell states, spatial disorganization, and disrupted intercellular communication.

Among the molecular regulators of these repair processes, microRNAs (miRNAs) act by controlling gene expression after transcription. These short non-coding RNAs regulate inflammation, angiogenesis, cell migration, fibroblast activation, extracellular matrix remodeling, and extracellular vesicle-mediated communication [14]. Significant dysregulation of miRNA expression profiles has been reported in both local wounds and the circulation of patients with DFUs [15]. Accordingly, exogenous supplementation or inhibition of selected miRNAs has shown therapeutic potential in experimental wound models [16]. Modern exosome engineering and biomaterial-based delivery strategies may further enhance miRNA stability, targeting, and multi-pathway intervention capacity [17,18,19,20,21,22].

However, most current studies still treat miRNAs as static biomarkers or isolated therapeutic targets [23] while insufficiently addressing the temporal and spatial abnormalities that distinguish DFUs from physiological wounds. Beyond the phase-level scale of inflammation, proliferation, and remodeling, a second temporal layer, circadian time, may also modulate the readiness of wound-resident and circulating cells to respond to the same miRNA signal at different times of day, consistent with recent epigenetic and circadian crosstalk frameworks proposed for diabetic neuropathy and diabetic wound repair [24]. These observations support examining how disrupted timing affects cell function and state transitions in DFUs, alongside changes in cell, cytokine, and miRNA abundance.

Timing is an established therapeutic variable in oncology and cardiovascular and respiratory medicine [25,26,27,28,29,30]. Applied to DFU miRNA biology, the relevant question therefore extends beyond which miRNA is altered to include the cell population, wound region, and repair window in which the alteration occurs. Building on the broader miRNA-circadian framework proposed for cutaneous repair [31], this review focuses on DFU-specific temporal dysregulation. The literature search and study-selection approach are summarized below. Section 2 explains the representative miRNAs used in the main text and the supporting examples in Table S1.

### Literature Search and Study Selection

Study design. This narrative review examines the roles of miRNAs across wound-healing stages and their dysregulation in diabetic foot ulcers. It integrates evidence from human studies, animal models, and cell experiments.

Literature search strategy. PubMed was searched for relevant literature available through 7 September 2026, with a primary focus on studies published during the preceding five years, from 7 September 2021 to 7 September 2026. Earlier publications were also included when relevant to wound-healing biology, miRNA mechanisms, or circadian regulation. Search terms included combinations of “diabetic foot” or “diabetic wound” with “microRNA” or “miRNA”. Additional terms covered wound healing, temporal regulation, healing phases and stages, circadian rhythms, BMAL1, PER2, extracellular vesicles, exosomes, delivery, mimics, inhibitors, MRG-110, and remlarsen. Relevant ClinicalTrials.gov records were checked on 6 September 2026.

Selection criteria. Publications were assessed by title and abstract, with full texts consulted where accessible. Primary human, animal, and cell studies were selected for their relevance to miRNA expression, wound-repair mechanisms, or therapeutic outcomes. Guidelines and reviews were included to provide clinical and biological context. Non-diabetic wound studies served as physiological comparators, and relevant non-wound studies provided mechanistic background. Duplicate and off-topic records were excluded. Reports lacking sufficient information on the study model or outcomes were not used as primary evidence for temporal patterns or therapeutic efficacy.

Evidence synthesis. Findings were organized by wound-healing stage, context-dependent miRNA function, proposed patterns of temporal dysregulation, circadian regulation, and therapeutic application. Interpretation considered the study population or model, sampling and treatment times, and measured outcomes. Observed expression changes were distinguished from inferred stage associations. Preclinical treatment effects, human pharmacodynamic findings, and clinical efficacy in DFUs were evaluated separately.

## 2. The Temporal Regulatory Architecture of miRNAs in Wound Healing

Three dimensions of time used in this review. Because the word “time” carries at least three distinct meanings in wound biology, we define our usage here and apply it consistently throughout the manuscript. (i) Chronological time denotes elapsed time from an explicitly specified reference event, such as injury or wound creation, ulcer onset, treatment initiation, or sample collection, as measured within a particular study and model (e.g., hours or days post-wounding or post-treatment). (ii) Biological wound-healing stage denotes the predominant functional state of the wound, defined by cellular composition and molecular activity rather than by the calendar. It includes inflammatory defense, inflammation-to-proliferation transition, tissue formation, or matrix remodeling. These programs may overlap spatially and temporally within the same wound. Acute experimental wounds often show an approximate but model-dependent correspondence between chronological time and biological stage, whereas chronic DFUs may retain persistent inflammatory features despite prolonged duration. Throughout this review, “wound-healing phase” and “wound-healing stage” refer to this biological dimension. (iii) Circadian time denotes phase within the endogenous approximately 24-h timing system that may modulate the responsiveness of wound-resident and circulating cells. It is distinguished from clock time or time of day when endogenous circadian phase was not directly assessed, and is addressed separately in Section 6. Where a cited study reports only chronological time, we preserve the original time points and their reference event and do not automatically re-label them as biological stages. Any mapping of such time points onto a wound-healing stage is identified as a model-specific inference.

Physiological cutaneous wound healing is often divided into inflammatory, proliferative, and remodeling phases, but these phases overlap and vary across wound regions. Their timing matters. Inflammation rises rapidly to defend and clear the wound, then declines sufficiently to permit epithelial migration, angiogenesis, fibroblast activation, granulation tissue formation, extracellular matrix deposition, and later matrix remodeling [32,33,34,35]. Recent spatiotemporal single-cell evidence further strengthens this view by showing that human wound repair is organized through coordinated changes in wound regions, cell states, and intercellular signaling over time, rather than through uniform whole-tissue changes alone [11]. Therefore, the main text of this review is anchored by representative phase-linked miRNAs that map onto key transitions: miR-155 and miR-146a for inflammatory activation and restraint, miR-132 and miR-21-5p for the inflammation-to-proliferation transition, miR-31 and miR-126 for epithelial and vascular repair, and miR-29 for matrix remodeling. We used the suffixes -3p and -5p when the mature strand can be identified from the cited study, its reported sequence, or the reagent used. Otherwise, we retained the source name and indicate that the mature arm was not specified. For miR-21, MIR21 gives rise to two distinct mature strands, miR-21-5p and miR-21-3p. The unsuffixed name miR-21 neither identifies the strand measured nor implies that both strands were measured together. We distinguished gene-level manipulations of MIR21/Mir21 from precursor-level manipulations involving pre-miR-21. For Ref. [36], we retained the source label miR-21-3p and marked it with a dagger (†) to indicate uncertainty in strand assignment, as explained in Table 1. Other miRNAs were retained as supporting examples and summarized in Table S1, whereas Table 2 integrates the major physiological and pathological miRNAs discussed in the core temporal framework. The physiological phase sequence, its major cellular events, and the representative miRNA anchors used in this review are integrated in Figure 1 and Table 2.

**Table 2:** Representative miRNAs in physiological wound-healing timing and DFU temporal dysregulation.

miRNA	Temporal Position	Physiological Evidence	Diabetic-Wound/DFU Evidence	Key Targets	Pathway	Stage Relevance
miR-155	Inflammatory activation/persistence	Mouse, day 10 [49]. Macrophage induction, in vitro [50,51].	Minipig time-course, elevated [38]. Human cross-sectional [52]. Human interventions, reduced after NPWT/PRF [53,54]. Animal inhibition [55,56].	SOCS1, SHIP1, FGF7	TLR/NF-κB; SOCS1/SHIP1; FGF7–epithelial repair	Inflammatory persistence.
miR-146a	Inflammatory feedback/resolution	Immune-cell feedback, in vitro [57].	db/db mouse, reduced, days 1, 3, 7 [37]. Minipig [38]. Deletion/delivery [58,59,60].	IRAK1, TRAF6	TLR/IL-1/TNF–NF-κB negative feedback	Inflammatory restraint.
miR-132	Inflammation-to-proliferation transition	Human acute wounds, rise, 24 h, peak, 1 week, fall, 1 month. Keratinocytes, in vitro [61].	Diabetic mouse intervention, miR-132-3p EVs improve repair [62].	HB-EGF	NF-κB restraint; EGFR/STAT3/ERK-associated keratinocyte growth	Resolution and growth.
miR-21-5p	Epithelial transition	Keratinocytes, in vitro, and mouse interventions, improved repair [47,48].	Not assessed [47,48].	TIMP3, TIAM1, PDCD4	TIMP3/TIAM1-associated migration [47]; PDCD4/TIMP3/MMP2-related repair [48]	Epithelial repair.
miR-21 (arm not specified)	Transition, context-dependent	Mouse, increased, days 1, 3, 7 [46]. Repair mechanisms [63,64,65].	Diabetic mouse, higher, days 1, 14, 21, lower, days 3, 7 [46].	PTEN, PDCD4, TIMP3, TIAM1	PTEN/PI3K-Akt; NF-κB; context-dependent	Phase mismatch.
miR-31	Proliferation/epithelial repair	Human wound edge, increased, days 1–7 [66]. Repair mechanisms [67,68].	Diabetic rat, day 7 [69]. Human cross-sectional/in vitro, reduced miR-31-5p [70].	EMP1, RASA1, SPRED1/2, SPRY4	NF-κB/STAT3 to Ras/MAPK-associated epithelial repair	Repair, delay unproven.
miR-126	Proliferation/angiogenesis	Vascular mechanisms, animal/in vitro [71,72].	Minipig, reduced [38]. Diabetic rat NPWT, increased, days 3, 5, 7, 9 [73].	SPRED1, PIK3R2	VEGF/MAPK-ERK and PI3K-Akt angiogenic signaling [74]	Vascular repair.
miR-29 family	Remodeling/matrix control	Mouse miR-29b, reduced, days 3, 7, 14, excisional nadir, day 7 [75].	Minipig miR-29a, rises in diabetic, falls in non-diabetic controls [38].	HSP47, COL1A1, COL3A1	TGF-β/Smad/CTGF regulation [76], collagen synthesis [75,77]	Matrix control.
miR-122-5p	Inflammatory persistence/impaired transition	Time-course unestablished [40].	Human cross-sectional and diabetic mouse day 14, increased. Separate intervention [40].	VEGF, FN1	NF-κB; MMP9/TNF-α and VEGF/fibrosis signaling	Inflammatory persistence.

Note: Stage relevance is inferred. Physiological wound studies are non-diabetic. Human [61,66] and animal day-series are expression time-courses, except serial treatment studies [53,54,73]. Days follow injury, but minipig days 0–15 [38] follow treatment baseline, with standard-care controls without APG. Diabetic expression comparisons use non-diabetic controls. Mechanistic evidence alone does not establish a time-course. Unspecified arms follow Section 2.

### 2.1. Inflammatory Phase: Activation, Defense, and Resolution

The inflammatory phase is a temporally programmed rise-and-resolution process, rather than a state that should be suppressed from the beginning. Immediately after injury, tissue damage, microbial signals, platelet-derived mediators, and danger-associated molecular patterns activate resident keratinocytes, fibroblasts, endothelial cells, and innate immune pathways [32,33]. Neutrophils are recruited early to remove pathogens and damaged material through phagocytosis, degranulation, reactive oxygen species production, protease release, and neutrophil extracellular trap formation [32,33,34]. Monocytes are subsequently recruited and differentiate into wound macrophages, which further amplify host defense by producing inflammatory cytokines and chemokines such as TNF-α, IL-1β, IL-6, CCL2, and CXCL8 [33,34,35]. This early inflammatory rise is necessary because insufficient activation can compromise microbial clearance and wound-bed preparation.

As repair progresses, however, the same inflammatory program must be actively restrained. Neutrophils undergo apoptosis or reverse migration, and macrophage-mediated efferocytosis of apoptotic neutrophils helps shift the wound environment from an inflammatory state toward a pro-resolving and reparative state [34,35]. This transition is accompanied by reduced NF-κB-driven cytokine signaling and increased production of mediators such as IL-10, TGF-β, *VEGF*, and PDGF, which support keratinocyte migration, fibroblast activation, angiogenesis, and granulation tissue formation [32,34,35]. Therefore, physiological inflammation has a biphasic logic: it must rise rapidly to defend and clear the wound, then decline in time to permit tissue formation. Failure of this switch, rather than inflammation itself, is a central feature of chronic wound stalling.

Within this inflammatory miRNA network, miR-155 is used as a representative activation-associated anchor. In primary macrophages, miR-155 is induced by Toll-like receptor ligands, TNF-α-associated inflammatory signaling, and interferon-related stimulation, identifying it as a common miRNA response to innate immune activation [50]. Functionally, miR-155 is enriched under M1-like inflammatory stimulation, and miR-155-deficient macrophages show reduced expression of inflammatory mediators, supporting its role in shaping the inflammatory macrophage transcriptional program [51]. Mechanistically, miR-155 can amplify inflammatory signaling by repressing negative regulators such as SOCS1 and SHIP1, whereas IL-10 suppresses TLR-induced miR-155 expression [78,79,80]. These studies establish an inducible inflammatory–amplification circuit more clearly than they establish a conserved wound-time trajectory.

Wound-healing studies provide a second layer of support. miR-155 expression increases in wounded skin, and genetic deletion of miR-155 improves dermal wound closure in mice, with a more reparative inflammatory milieu and increased type I collagen deposition [49]. In diabetic wound models, miR-155 inhibition accelerates wound closure, reduces inflammatory-cell infiltration and inflammatory cytokine expression, and improves neovascularization and collagen organization [55]. Another diabetic skin study showed that miR-155 is upregulated under diabetic conditions and that topical miR-155 inhibition restores FGF7 expression, reduces wound inflammation, enhances re-epithelialization, and accelerates closure [56]. Human DFU data further support clinical relevance: miR-155 expression is increased in peripheral blood and wound-margin tissue of patients with DFU and is associated with ulcer severity, lower healing rate, and longer healing time [52]. More recently, negative-pressure wound therapy was reported to reduce miR-155 expression in DFU granulation tissue, and the degree of miR-155 downregulation correlated with wound-healing rate [53]. Taken together, these studies support miR-155 as a representative inflammatory-drive miRNA in wound healing, while also highlighting the need for a counter-regulatory mechanism that restrains inflammation during the transition toward repair.

miR-146a provides a complementary feedback-regulatory example. Mechanistically, inflammatory stimulation through the TLR-, IL-1-, and TNF-related pathways induces miR-146a in an NF-κB-dependent manner, after which miR-146a represses upstream adaptor molecules such as IRAK1 and TRAF6 to limit excessive NF-κB signaling [57,81]. In db/db mouse wounds, reduced miR-146a after injury was associated with increased IRAK1, TRAF6, NF-κB, IL-6, and MIP-2 expression; mesenchymal stem cell treatment restored miR-146a, attenuated inflammatory gene activation, reduced inflammatory-cell accumulation, and improved closure [37]. miR-146a deficiency also delayed healing in normal and diabetic mice, with greater neutrophil/macrophage infiltration and inflammatory signaling [58]. Cerium oxide nanoparticle-conjugated miR-146a and miR-146a-loaded engineered exosomes further reduced inflammatory signaling and promoted diabetic wound repair in preclinical models [59,60]. Conceptually, miR-155 and miR-146a illustrate inflammatory amplification and counter-regulatory feedback. Notably, these data derive from different models and sampling schedules, and whether this pair follows a matched two-miRNA time course in human wounds remains to be verified.

### 2.2. Inflammation-to-Proliferation Transition: Resolution and Reparative Reprogramming

The transition from inflammation to proliferation is not a canonical healing phase but a recognized regulatory transition window. Landén et al. described the shift from inflammation to tissue regeneration as one of the most critical steps in skin wound healing, and inefficient transition between these two phases has been linked to hard-to-heal ulcers and abnormal repair [35]. Ellis et al. similarly emphasized that the key transition point lies between the inflammatory and proliferative phases, and that chronic wounds often fail to complete this switch [82]. This concept has recently been strengthened by cell-state studies. A 2024 Nature Communications study showed that keratinocyte progenitor cells undergo an inflammatory-to-proliferative state transition during wound healing, indicating that this switch is not restricted to immune cells but also occurs within epithelial repair programs [83]. Human spatiotemporal single-cell data further show that normal wound repair depends on coordinated shifts across inflammatory, epithelial, fibroblast, and vascular compartments, whereas chronic wounds and DFUs are associated with failed keratinocyte migration and impaired inflammatory responses [11]. Thus, this transition window represents a biologically meaningful handoff from defense and clearance to tissue formation.

Among the miRNAs, miR-132 provides particularly direct evidence that this transition can be regulated by miRNA dynamics. In human skin wounds, miR-132 is upregulated during the inflammatory phase, is mainly expressed in epidermal keratinocytes, and reaches its peak during the subsequent proliferative phase [61]. Functionally, miR-132 suppresses keratinocyte production of inflammatory chemokines through NF-κB-related signaling while promoting keratinocyte growth through the STAT3 and ERK pathways [61]. In mouse and human ex vivo wound models, blockade of miR-132 delayed wound healing, with aggravated inflammation and insufficient keratinocyte proliferation [61]. Therefore, miR-132 is an important mechanistic proof-of-concept: miRNA expression can actively facilitate the transition from inflammation to proliferation, rather than merely reflecting phase progression.

Studies of miR-21 link inflammatory resolution, macrophage plasticity, epithelial–immune communication, and early stromal repair. During wound resolution, efferocytosis of apoptotic cells by macrophages induces source-reported miR-21 (mature arm not specified in the source study), which suppresses inflammatory signaling through targets such as PTEN and PDCD4 and promotes an IL-10-associated anti-inflammatory response [63]. These findings place the reported miR-21 activity on the resolution side of the transition, where macrophages move away from inflammatory amplification and toward a reparative state.

Further evidence links miR-21 to reparative reprogramming. Sinha et al. showed that injury-site myeloid cells can convert into fibroblast-like cells within granulation tissue and that this transition is impaired in diabetic wound tissue [64]. Keratinocyte-specific Mir21 deletion reduced this conversion in wounds, whereas myeloid-specific deletion did not impair conversion in vivo. Keratinocyte-derived extracellular vesicles carried miR-21 to myeloid cells, supporting a role for epithelial–myeloid communication [64]. These experiments did not establish the contribution of each mature strand. Keratinocyte-derived exosomal communication with macrophages has also been shown to contribute to cutaneous wound repair, supporting the view that epithelial–immune crosstalk is an active driver of transition rather than a passive bystander event [84].

miR-21 also participates in the tissue-formation side of the transition, and this is the best strand-resolved component of the evidence. In keratinocytes, miR-21-5p, verified by Northern blot against the mature miR-21-5p sequence, promotes migration and re-epithelialization by targeting TIMP3 and TIAM1 [47], and a sequence-traceable miR-21-5p mimic (mirVana product MC10206) reproduced pro-reparative keratinocyte effects in nanocarrier-based delivery [48]. In mouse skin wounds, source-reported miR-21 (mature arm not specified) is induced after injury, and miR-21 inhibition delays wound closure, reduces collagen deposition, and impairs multiple aspects of the repair process. The same study used a pre-miR-21 expression plasmid for gain-of-function, so the manipulations act at precursor and mature levels rather than on a single confirmed strand [65]. These studies link miR-21 to inflammatory resolution and early tissue formation, with different levels of strand identification.

Compared with miR-132, miR-21 is not the most direct or exclusive regulator of this transition. Its effects vary by cell type, wound stage, metabolic background, and tissue endpoint [46,85], and are examined in Section 3. miR-132 therefore provides the strongest evidence that the transition window is miRNA-regulated, whereas miR-21 acts as a context-dependent bridge. Other transition-related miRNAs, including miR-497, miR-34 family members, and miR-125b, are summarized in Table S1.

### 2.3. Proliferative Phase: Re-Epithelialization and Vascularized Granulation

After the wound has moved beyond immune-dominant clearance, the proliferative phase is defined by coordinated tissue formation rather than by cell proliferation alone. Its two most visible requirements are re-epithelialization and vascularized granulation tissue formation. Keratinocytes at the wound edge loosen stable adhesions, migrate over the provisional matrix, proliferate behind the migrating front, and later re-establish epithelial stratification and barrier continuity. In parallel, fibroblasts expand within the wound bed, deposit provisional extracellular matrix, and cooperate with endothelial cells to form a vascularized granulation tissue that can sustain repair [11,32]. Recent human spatiotemporal single-cell data support this coordinated view: day-7 acute wounds show proliferative-phase programs such as cell division and extracellular matrix organization, while comparison with chronic wounds and DFUs links failed keratinocyte migration with impaired inflammatory and repair responses [11]. Thus, the proliferative phase should be interpreted as a timed readiness state of epithelial, stromal, and endothelial compartments.

miR-31 is used here as the representative epithelial-readiness miRNA. In an in vivo human skin wound model, miR-31 was gradually upregulated in wound-edge keratinocytes from the inflammatory phase through the proliferative phase, and miR-31 overexpression promoted proliferation and the migration of human primary keratinocytes, whereas miR-31 inhibition had the opposite effect [66]. Mechanistically, EMP1 was identified as a direct target of miR-31, linking miR-31 induction to keratinocyte motility and wound-edge activation [66]. Mouse loss-of-function data further strengthen this interpretation. miR-31 is induced in wound-edge keratinocytes by inflammatory NF-κB and STAT3 signaling, reaches high expression during the proliferative phase, and promotes keratinocyte proliferation and migration by repressing negative regulators of Ras/MAPK signaling, including Rasa1, Spred1, Spred2, and Spry4 [67]. In a related mucosal-healing model, ectopic miR-31 expression enhanced keratinocyte proliferation and migration in vitro and accelerated skin wound closure in vivo [68]. Together, these studies support miR-31 as a phase-linked epithelial mediator that translates earlier inflammatory signals into re-epithelialization.

Epithelial repair failure in diabetic wounds is not determined by miR-31 alone but reflects a broader failure of timely miRNA induction. In diabetic rat wounds, delayed healing was associated with a downregulated miRNA signature at day 7 after injury, together with reduced Dicer expression and impaired keratinocyte migration after Dicer inhibition [69]. miR-31 therefore marks epithelial readiness within a wider diabetic defect in wound-edge activation.

The second axis of the proliferative phase is vascular readiness, for which miR-126 is the most suitable anchor. miR-126 is enriched in endothelial cells and is required for vascular integrity, endothelial migration, and angiogenic signaling. Genetic loss of miR-126 impairs endothelial proliferation, migration, vascular integrity, and angiogenesis in vivo [71]. Mechanistically, miR-126 promotes VEGF-related angiogenic signaling by repressing negative regulators such as SPRED1 and PIK3R2, thereby enhancing MAPK/ERK and PI3K-related signaling in endothelial cells [72]. These findings make miR-126 a biologically coherent marker of endothelial competence during granulation tissue formation.

Diabetic wound studies provide additional support for this vascular interpretation. In diabetic rat wounds treated with negative-pressure wound therapy, miR-126 expression increased on days 3, 5, 7, and 9, in parallel with greater capillary and arteriolar density; miR-126 upregulation was also associated with increased VEGF and phosphorylated ERK expression and reduced SPRED1 expression [73]. Circulating miR-126 levels correlated with angiogenesis indices, suggesting that miR-126 may reflect vascular repair activity in diabetic wounds [73]. This does not establish miR-126 as the only angiogenic miRNA, but it supports its use as a representative vascular-readiness miRNA within the temporal framework.

Other proliferative-phase miRNAs, including miR-205, the miR-200 family, miR-210, miR-195-5p/miR-205-5p, and miR-199-5p, provide useful supporting examples for epithelial plasticity, angiogenesis, extracellular vesicle signaling, and neurovascular repair, and are summarized in Table S1. In the main narrative, miR-31 and miR-126 are retained as paired anchors because they capture the two essential outputs of the proliferative phase: timely epithelial coverage and the formation of a vascularized wound bed. Once these programs have produced coverage and granulation tissue, the central regulatory problem shifts from tissue formation to matrix maturation and remodeling.

### 2.4. Remodeling Phase: Matrix Maturation and Functional Tissue Repair

Once epithelial coverage and vascularized granulation tissue have been established, the central task of wound healing shifts from tissue formation to tissue quality. The remodeling phase is characterized by gradual replacement and reorganization of provisional extracellular matrix, maturation of collagen architecture, regression of unnecessary vessels and cells, and restoration of tensile strength [32,86]. Collagen III-rich early matrix is progressively replaced by more organized collagen I, while matrix metalloproteinases and their inhibitors regulate collagen degradation, deposition, and alignment [86]. Therefore, remodeling should not be understood as a passive final stage. It is an active quality-control process that determines whether wound closure results in functional tissue, fragile recurrent breakdown, or excessive scar formation.

The miR-29 family is used here as the representative matrix-remodeling miRNA because it connects miRNA regulation to collagen biosynthesis, fibroblast activity, and scar-related matrix balance. miR-29 family members function as negative regulators of extracellular matrix genes, including collagens and other matrix-associated transcripts. In skin wound models, Zhu et al. showed that miR-29b overexpression reduced collagen biosynthesis by inhibiting heat shock protein 47, a collagen-specific molecular chaperone, supporting a mechanistic role for miR-29b in controlling collagen production during skin repair; notably, in the same model, local miR-29b delivery also suppressed wound angiogenesis, a finding directly relevant to the temporal framework discussed below [75]. This makes miR-29 biologically suited to the remodeling phase, where matrix deposition must be refined rather than simply maximized.

Additional experimental and translational evidence supports this collagen-regulatory role. In a thermal injury model, miR-29b treatment promoted wound healing while reducing excessive scar formation through inhibition of the TGF-β1/Smad/CTGF pathway [76]. A miR-29 mimic, remlarsen, repressed extracellular matrix gene expression and fibroplasia across preclinical skin models and showed pharmacodynamic activity in incisional skin wounds in healthy human volunteers [77], establishing miR-29 as an anti-fibrotic and matrix-modulating principle. More recent human skin-cell work also shows that miR-29 regulates both epidermal and mesenchymal functions, including cell-to-matrix adhesion and extracellular matrix-related target programs in keratinocytes and fibroblasts [87]. Together, these studies position miR-29 as a regulator of matrix quantity, matrix organization, and cell–matrix interaction.

In a temporal framework, the key point is not that miR-29 should always be increased or suppressed. Because miR-29 restrains collagen and matrix-associated programs, premature or excessive miR-29 activity during early granulation could theoretically limit necessary matrix deposition, whereas insufficient miR-29 activity during later remodeling may permit excessive fibroplasia or disorganized matrix accumulation. This context-dependent logic is particularly relevant to DFUs, where closure alone does not guarantee durable repair. The therapeutic endpoint should be a stable, vascularized, mechanically competent tissue architecture rather than rapid but fragile epithelial coverage.

Other remodeling-related or multi-phase miRNAs, including miR-182-5p, miR-34 family members, miR-205, and the miR-200 family, are retained as supporting examples in Table S1. In the main temporal narrative, miR-29 is therefore used to close the physiological sequence: miR-155 and miR-146a frame inflammatory activation and restraint, miR-21 and miR-132 illustrate the inflammation-to-proliferation transition, miR-31 and miR-126 represent epithelial and vascular readiness, and miR-29 anchors the shift from tissue formation to matrix maturation. This sequence supports the central argument of the review: effective wound repair depends on whether a miRNA’s activity occurs within the right biological window, extending beyond its mere presence.

## 3. Context-Dependent Roles of miRNAs in Wound Healing

The phase-resolved framework should not assign any miRNA a fixed pro-healing or anti-healing identity. Its observed effect depends on cell type, sampling or treatment time, species and tissue model, dose and delivery system, and tissue-level endpoint. Hyperglycemia, chronic inflammation, oxidative stress, impaired perfusion, and matrix disorganization further modify these relationships in DFUs. Table 1, Table 2 and Table 3 identify the population or model and study design supporting each entry. We distinguish human time-course and cross-sectional observations, human repeated-measure interventions, animal time-course or intervention studies, and in vitro experiments. Inferred stage assignments are identified separately. A treatment response or cross-sectional difference is not treated as a directly observed healing trajectory.

miR-21 is reported both as a pro-reparative and as a pro-inflammatory molecule. Macrophage efferocytosis induces source-reported miR-21, which drives inflammatory resolution through PTEN and PDCD4 [63], and keratinocyte miR-21-5p promotes migration and re-epithelialization [47,48], with additional source-reported, arm-unspecified support [65]. In diabetic wound macrophages, in contrast, LPS and high glucose induce source-reported miR-21 in a dose- and time-dependent manner and amplify inflammatory markers through PTEN-related NOX2 and PI3K signaling [46]. The studies differ in how clearly they identify the mature strand: keratinocyte studies resolved miR-21-5p [47,48], whereas the macrophage and wound-trajectory studies reported miR-21 without specifying the mature arm [46,63]. These findings should therefore be compared by cell type, wound-healing stage, metabolic exposure, and the available strand information.

miR-29 exposes a genuine disagreement about the direction of correction. Where matrix deposition is excessive, suppressing collagen synthesis is beneficial: miR-29b limits scarring through TGF-β1/Smad/CTGF signaling [76], and remlarsen represses extracellular-matrix expression and fibroplasia in incisional wounds in healthy volunteers [77]. Where matrix generation is already deficient, the same suppression carries risk: in the model that established miR-29b-mediated HSP47 repression, local miR-29b delivery also suppressed wound angiogenesis [75], and in a minipig study, miR-29a declined in non-diabetic controls but rose in diabetic controls over the treatment-observation period. Autologous platelet-rich gel improved healing and lowered miR-29a relative to diabetic standard-care controls [38]. These findings suggest that the effect of miR-29 depends on existing matrix deposition and vascular repair. Studies of miR-29-directed interventions should therefore assess baseline matrix deposition and monitor angiogenesis for both benefit and harm.

Species and delivery platform further constrain translation. Human and mouse skin differ in architecture, cellular composition, and repair kinetics, and direct cross-species comparison cautions against simple extrapolation from murine trajectories [11]. Naked oligonucleotides, chemically modified mimics or inhibitors, extracellular-vesicle cargo, hydrogels, and nanoparticles also create different cellular exposures. Dose- and time-dependent target derepression by a locked-nucleic-acid miR-92a inhibitor illustrates that the formulation is part of the biological intervention [88]. Preclinical findings should therefore be classified as mechanistic or translational support according to the model used, not as direct evidence of human DFU efficacy.

Context dependence thus sharpens the temporal framework. Interpretation should identify the producing or receiving cell, time and location of action, species and disease model, achieved exposure, and tissue outcome. This context helps interpret the proposed patterns discussed next, delayed or insufficient induction, aberrant persistence, and temporal inversion. Expression direction alone cannot distinguish these patterns.

**Table 3:** Stage-aware miRNA therapeutic strategies: stage markers and spatiotemporally controlled delivery.

miRNA	Intervention/Delivery	Proposed Context	Candidate Markers	Mechanism/Target	Functional Effect	Evidence
**Restrain persistent inflammation**						
miR-155	Anti-miR, MSC EVs	Persistent inflammation	High IL-6/TNF-α and miR-155	Reduces IL-6/TNF-α, restores SOCS1/SHIP1 restraint.	Less inflammation, greater keratinocyte migration and collagen.	C. Diabetic mice, in vitro [89].
miR-125b	Mimic, ADSC EVs	Inflammatory infiltration	High TLR4/M1 dominance	Reduces TLR4/IL-6/DLL4, increases VEGF/TGF-β1.	Less inflammatory infiltration.	C. Diabetic rats, HUVECs [90].
miR-26b-5p	Mimic, PRP EVs	Inflammation, persistent NETs	Excess NETs; high MMP-8	MMP-8 inhibition.	Less inflammation and NET formation.	C. Diabetic mice, NET assays. Human cross-sectional [91].
miR-146a-5p	Mimic, BMSC EVs	Transition, insufficient feedback, Pattern 1	Low miR-146a; high IRAK1/TRAF6	TRAF6 inhibition, M2 polarization.	↑ re-epithelialization and neovascularization	C. Diabetic mice, macrophages [92].
**Restore angiogenesis**						
miR-126-3p	Mimic, IFN-γ-pretreated BMSC EVs	Tissue formation, poor angiogenesis	Low miR-126; poor granulation vascularity	SPRED1 inhibition, Ras/ERK activation.	↑ endothelial proliferation, migration, tube formation	C. Diabetic mice, endothelial cells [93].
miR-106a-5p	Indirect inhibition, circ_0001052-enriched ADSC EVs	Tissue formation, anti-angiogenic activity	Low FGF signaling	Reduces miR-106a-5p activity, activates FGF4/p38 MAPK.	Greater angiogenesis.	C. Diabetic mice, HUVECs [94].
miR-125b	Mimic, ADSC EVs	Tissue formation, poor vascular repair	Low VEGF/TGF-β1	↑ VEGF and TGF-β1	↑ endothelial proliferation, migration and angiogenesis	C. Diabetic rats, HUVECs [90].
miR-138	Mimic, ADSC EVs	Tissue formation, poor angiogenesis	Suppressed PI3K/Akt & ERK	SIRT1/PTEN, restores PI3K/Akt and ERK signaling.	↑ endothelial function; neovascularization	C. Diabetic rats, endothelial cells/fibroblasts [95].
anti-miR-92a-3p (MRG-110)	LNA anti-miR, systemic/local	Tissue formation, anti-angiogenic activity	High miR-92a-3p; low endothelial recovery	miR-92a-3p inhibition, angiogenic target derepression.	Preclinical angiogenesis and revascularization.	B. Healthy-human blood PD [96]. C. db/db mice, non-diabetic pigs [88]. Registry [97].
**Support proliferation/re-epithelialization**						
miR-132-3p	Mimic, Insig1-enhanced BMSC EVs	Tissue formation, poor epithelial proliferation	Low proliferative drive	Regulates MMP-9 and PDGF	Faster wound closure.	C. Diabetic mice, in vitro [62].
**Remodeling-oriented matrix control**						
miR-128-1-5p	Mimic, ADSC EVs	Remodeling, excess fibrosis	Excess α-SMA/collagen I; scarring risk	Targets TGF-β1/SMAD.	Reduced α-SMA, collagen I and fibrosis.	C. Diabetic rats, fibroblasts [98].
miR-29b (remlarsen/MRG-201)	Synthetic mimic, intradermal	Remodeling, excess/phase-inappropriate fibrosis	Low miR-29; excess collagen deposition	Restores miR-29, reduces COL1A1 and COL3A1.	Reduced collagen biomarkers and fibroplasia, healthy wounds.	B. Healthy-human wound PD. C. Non-DFU mice/rats/rabbits [77]. Registries [99,100].

Note: Stage contexts and candidate markers are exploratory, not validated staging tools or dosing schedules. A, human DFU efficacy. B, human pharmacodynamics (PD). C, preclinical evidence. No row establishes Tier A efficacy of a defined miRNA mimic or inhibitor. Animal evidence is interventional, cell assays are in vitro. Human cross-sectional data provide context, not efficacy. EV, extracellular vesicle. Registry check, 6 September 2026. All three trials completed. No results posted for NCT03603431 or NCT02603224. NCT03601052 results first posted 19 July 2021. MRG-110 systemic PD [96] is separate from the local wound trial [97]. ↑ indicates an increase relative to the comparator in the cited study.

## 4. miRNA Temporal Dysregulation in DFU: Three Interpretive Patterns

We propose three interpretive patterns, delayed or insufficient induction, aberrant persistence, and temporal inversion. They form a conceptual framework for organizing heterogeneous human, animal, and in vitro evidence. The framework relates these findings to biological wound-healing stage while retaining each study’s sampling times and model. The proposed patterns require validation in longitudinal human DFU studies.

### 4.1. DFU Chronicity as a Wound-State Coordination Disorder

Compared with acute physiological repair, DFU healing fails in a microenvironment shaped by hyperglycemia, hypoxia, oxidative stress, neuropathy, vascular insufficiency, infection risk, persistent inflammation, and epigenetic memory—the metabolic-inflammatory context in which every miRNA signal discussed below operates [24,101]. These stresses can alter local and circulating non-coding RNA signals, including extracellular miRNAs. However, DFU chronicity is not adequately explained by the absence of a single reparative miRNA; it is increasingly understood as a loss of coordination between wound stage, wound region, cell state, and molecular signal. Figure 2 summarizes the DFU wound environment and major cellular consequences. Table 1 organizes the miRNA findings into the three interpretive patterns.

A recurring source of apparent contradiction is that the same miRNA is scored as increased or decreased depending on the comparison used. Three axes are commonly conflated: a within-wound temporal trajectory, a diabetic-versus-non-diabetic contrast at matched time points, and a treatment response. In the diabetic minipig series, miR-146a rose across the healing course yet remained below non-diabetic levels at matched times, while miR-29a moved in opposite directions in non-diabetic and diabetic wounds [38]. Whether an observation reads as insufficient induction, aberrant persistence, or temporal inversion therefore depends on which axis was measured, and reported directions cannot be pooled across studies without specifying it.

Single-cell and spatial studies provide the cellular context for this framework. Theocharidis et al. showed that healing and non-healing DFUs differ in cell-state composition and spatial organization, including healing-associated fibroblasts enriched toward the wound bed and distinct macrophage states [12]. The human spatiotemporal roadmap linked chronic wounds, including DFUs, to failed keratinocyte migration and impaired inflammatory responses [11]. A 2026 secondary analysis of public transcriptomic datasets reported collagen-centric signaling, altered immune-cell abundance, and dysfunction in monocyte/macrophage and endothelial compartments [13]. None of these studies directly measured longitudinal miRNA trajectories; rather, they identify the changing cell states and tissue regions within which miRNA signals must be interpreted.

### 4.2. Pattern 1: Delayed or Insufficient Induction of Reparative Programs

The first pattern is delayed or insufficient induction. In this pattern, the wound does not activate epithelial, vascular, or stromal repair programs during the window in which proliferation, migration, and granulation tissue formation are needed. A reparative signal may be too weak or may appear too late to meet the needs of cells in the wound bed.

miR-31 illustrates this pattern across mechanism, cell behavior, and tissue repair. In physiological human skin wounds, miR-31 is low in intact skin but gradually increases in wound-edge keratinocytes from day 1 to day 7 after injury, across the inflammatory and proliferative periods described in this human wound model [66]. Mechanistically, miR-31 promotes keratinocyte proliferation and migration by suppressing epithelial membrane protein 1 (EMP-1) [66], and subsequent work showed that inflammatory NF-κB/STAT3 signaling induces miR-31 to activate Ras/MAPK-related re-epithelialization programs through targets including Rasa1, Spred1, Spred2, and Spry4 [67]. At the cellular level, this places miR-31 at the wound-edge keratinocyte migration front; at the tissue level, it supports timely re-epithelialization.

In diabetes, the same epithelial program appears insufficiently engaged. Diabetic rat wounds showed broad miRNA downregulation at day 7 relative to normal wounds, when diabetic wounds remained larger and inflamed [69]. DFU-related tissue and high-glucose cell models also report reduced miR-31-5p in association with impaired viability and migration through an SNHG16/miR-31-5p axis [70]. These observations are consistent with inadequate activation of an epithelial migration program. Because physiological and diabetic trajectories were not measured in one matched human cohort, the evidence supports insufficient induction more strongly than a formally demonstrated delay. Additional vascular evidence comes from studies of miR-126-3p-enriched extracellular-vesicle signaling [93,102].

miR-210 is also interpreted as an example of insufficient induction, as discussed in Section 5 and summarized in Table 1.

### 4.3. Pattern 2: Aberrant Persistence of Inflammatory or Anti-Reparative Signals

The second pattern is aberrant persistence. Here, an inflammatory or anti-reparative program remains active after it should have been restrained. This distinction matters: early inflammation is necessary for host defense and tissue cleanup, but persistent unproductive inflammation prevents a transition toward proliferation. miR-155 provides a representative example. It is upregulated in diabetic skin, and topical miR-155 inhibition restored FGF7 expression, reduced inflammation, and improved diabetic wound closure in experimental models [56]. A later study further showed that M1 macrophage-derived exosomal miR-155-5p suppresses endothelial migration and tube formation by targeting GDF6, while local antagomiR delivery promoted angiogenesis and accelerated repair in db/db mice [103].

Dynamic profiling in a diabetic Bama minipig wound model documented higher miR-155 in diabetic than non-diabetic controls over study days 0–15 from the treatment-observation baseline, with lower levels after autologous platelet-rich gel treatment [38]. Mechanistic studies add complementary support: miR-155 suppresses FGF7-associated re-epithelialization [56], and macrophage-derived exosomal miR-155-5p impairs endothelial migration and tube formation through GDF6 [103]. Together, these observations link persistent inflammatory signaling to reduced vascular and epithelial repair.

Human DFU evidence supports the same interpretation. Elevated miR-155 has been observed in DFU wound-margin tissue compared with non-diabetic wounds, and higher miR-155 levels were associated with Wagner grade. After 15 days of platelet-rich fibrin intervention, miR-155 levels decreased, while HIF-1α, VEGF expression, and vascular density increased in wound-margin tissue [54]. These observations are consistent with aberrant persistence, although the treatment-associated changes do not establish an untreated human DFU trajectory. Additional studies provide complementary evidence from wound tissue, macrophage-derived vesicles, endothelial models, and circulating miRNAs [40,43,44,46,104,105].

Complementary evidence comes from a recent double-blind pilot trial of topical folinic acid in chronic DFUs (*n* = 10). After 12 weeks, the folinic-acid group showed greater ulcer-area reduction than the control. Keratinocyte proteomic analysis identified reduced HMGB1 and activated IL-1β, together with lower activating phosphorylation of SAPK/JNK and p38 MAPK [106]. Genome-wide methylation analysis also identified reduced methylation at regulatory sites bioinformatically assigned to anti-inflammatory miRNAs predicted to target MAPK pathways [106]. Because miRNAs were not directly measured and the sample was small, the study provides exploratory human evidence linking wound improvement to epigenetic changes that may involve anti-inflammatory miRNA regulation.

### 4.4. Pattern 3: Temporal Inversion or Phase-Inappropriate Expression

The most time-resolved example available is source-reported miR-21, with the mature arm not specified in the original study: relative to non-diabetic wounds, diabetic mouse wounds showed higher miR-21 at day 1 and days 14 and 21, but lower expression during the day-3-to-7 window (the mid-proliferative window of the non-diabetic comparator) [46]. This matched-time-point profile comes from a single mouse dataset and should be regarded as model-specific evidence of inversion, not an established human DFU trajectory. A separate study reported reduced miR-21-3p† at 3 h and 24 h after high-glucose exposure in fibroblasts and on days 4 and 7 after wounding in db/db mice [36]. The source’s strand assignment is uncertain, as explained in Table 1.

In that study, the reported miRNA intervention suppressed SPRY1, improved fibroblast proliferation, reduced apoptosis, increased collagen III, bFGF and VEGF expression, and accelerated wound closure in db/db mice [36]. These findings support a reparative effect of the tested preparation, but do not establish a confirmed miR-21-3p-specific mechanism. Comparison with the macrophage findings [46] and strand-resolved keratinocyte studies [47,48] therefore requires attention to cell type, experimental conditions, and strand identification.

## 5. Integrated Pathology: Angiogenesis Impairment

Angiogenesis illustrates how temporal dysregulation becomes tissue pathology. Vascularized granulation normally requires a coordinated sequence of hypoxia sensing, endothelial activation, macrophage and fibroblast support, extracellular-matrix organization, and vessel maturation. Hyperglycemia, AGEs, oxidative stress, chronic inflammation, and impaired perfusion can disturb this sequence through both intracellular miRNA changes and extracellular-vesicle-mediated transfer. The resulting defect is therefore broader than reduced VEGF availability: molecular timing errors propagate across endothelial, immune, stromal, and matrix compartments. Representative pathways are integrated in Figure 2 and Table 1.

Human DFU endothelial studies define the phenotype that a temporal miRNA model must explain. Dermal microvascular endothelial cells isolated from DFUs showed impaired tube formation compared with healthy controls, while bulk and single-cell RNA sequencing identified downregulated angiogenic programs and RAB17 as a candidate regulator [107]. A separate single-cell study resolved inflammatory and angiogenesis-related endothelial states and linked SH3BGRL3 experimentally to endothelial proliferation, migration, and angiogenic capacity under high glucose [108]. These studies do not establish miRNA causality, but they identify the cell-level deficit: DFU endothelial cells are shifted away from coordinated migration, tube formation, and repair-associated activation. They therefore provide a phenotypic reference against which proposed miRNA timing mechanisms should be judged.

miR-126 provides treatment-associated temporal evidence, and two time courses appear to run in opposite directions. In diabetic rat wounds receiving negative-pressure wound therapy, wound and plasma miR-126 increased at days 3, 5, 7, and 9 alongside capillary and arteriolar density, circulating levels correlated with both vascular measures, and day-9 tissue showed lower SPRED1 with higher VEGF and p-ERK [73]. In diabetic minipig wounds receiving standard care without autologous platelet-rich gel (APG), miR-126 declined over study days 0–15 from the treatment-observation baseline [38]. APG treatment increased miR-126 relative to diabetic standard-care controls. These studies differ in species and treatment, so they should not be read as opposing trajectories from the same model.

A second mechanism is inadequate induction of hypoxia-responsive miRNAs. High glucose blunted hypoxia-induced miR-210 in vitro and in human and mouse diabetic wounds; restoring miR-210-3p reduced oxygen consumption and reactive oxygen species, activated glycolysis, improved migration, and accelerated healing in diabetic mice but not in non-diabetic mice [39]. Deficient miR-210 induction therefore reflects impaired metabolic readiness during early vascular adaptation rather than lower expression of one molecule, and failure to activate this program delays progression toward vascularized granulation tissue even when hypoxic stimuli are present. In the minipig study, miR-210 increased over time in non-diabetic wounds, whereas no significant temporal change was detected in diabetic control wounds [38]. Together with the evidence of impaired hypoxia-induced miR-210 expression [39], this supports its interpretation as insufficient induction (Pattern 1) rather than temporal inversion.

Pathological extracellular-vesicle cargo provides a complementary route. DFU wound-fluid vesicles enriched in miR-195-5p and miR-205-5p suppressed VEGFA, endothelial tube formation, and neovascularization [109]. AGE-stimulated macrophage vesicles likewise impaired diabetic repair, with vesicular miR-ERIA inhibiting endothelial migration and tube formation through helicase with zinc finger 2 [110]. Together, these studies provide a mechanism–cell–tissue chain: diabetic inflammation and AGEs reshape vesicle cargo; antiangiogenic signals are transferred to endothelium; endothelial migration, VEGFA signaling, and tubular organization are suppressed; and the wound bed fails to build an adequate vascular network.

These pathological vesicles sit alongside engineered exosomes that deliver miR-146a or miR-17-5p and promote diabetic wound repair [22,60]. The apparent paradox resolves once donor-cell state and cargo are treated as the operative variables: a vesicle is a carrier whose effect is shaped by the program of the producing cell and by what it has been loaded with, rather than by vesicle identity as such. Comparisons across laboratories should therefore begin with cellular source, induction state, and cargo profile, a constraint that also applies to clinical vesicle products whose active cargo has not been assigned to a defined miRNA [111].

Conversely, inhibiting antiangiogenic miRNAs or restoring reparative signals supports vascular repair, although the evidence remains mainly preclinical. The miR-92a inhibitor MRG-110 increased granulation, angiogenesis, perfusion, and closure in db/db mouse and normal pig wounds and derepressed proangiogenic targets including ITGA5 [88]. A first-in-human study subsequently demonstrated dose- and time-dependent miR-92a-3p suppression and target derepression, including ITGA5 and CD93, in healthy adults [96]. miR-17-5p-enriched small extracellular vesicles in GelMA also improved endothelial proliferation, migration, tube formation, angiogenesis, collagen deposition, and diabetic wound healing through PTEN and p21 in a preclinical model [22]. A systematic review of VEGF stimulation found encouraging in vivo trends but unresolved dosing, release, and mechanism [112]. Angiogenic miRNA therapy must therefore be matched to wound region, cell type, dose, and repair phase, and its DFU efficacy confirmed in randomized controlled trials.

## 6. Circadian Regulation and Wound Repair

The preceding sections define DFU chronicity through phase-level miRNA timing: reparative signals may be delayed, inflammatory programs may persist, and otherwise useful miRNAs may appear in the wrong context. Circadian biology adds a separate temporal dimension by potentially modifying the readiness of wound-resident and circulating cells to respond to the same signal across the day; it does not replace the wound-phase framework. Human skin shows population-level transcript rhythms, clock-gene activity in epidermal samples and explants, and context-dependent changes in rhythm amplitude or phase, including in sun-exposed skin [113,114,115,116]. These data establish biological plausibility; whether rhythms persist, shift, or collapse in chronic ulcers, and whether a DFU-specific circadian mechanism exists, are important questions for future research. The circadian system and core feedback loop are outlined in Figure 3.

Functional studies support timing-dependent repair. Fibroblast-intrinsic circadian actin dynamics regulate cell mobilization, and associated human burn data suggest that injury time may influence healing speed [117]. Environmental disruption by dim light at night delayed dermal repair in mice [118], while circadian disruption was associated with impaired epithelialization in diabetic mouse wounds [119]. Collectively, these findings suggest that circadian disruption may affect fibroblast mobilization, keratinocyte repair, oxidative-stress handling, and inflammatory tone within the wound microenvironment. These processes overlap directly with the phase-level failures described above, although their relevance to DFUs remains largely preclinical.

The cutaneous clock is organized around CLOCK–BMAL1 activation of clock-controlled genes, including PER and CRY, followed by PER/CRY-mediated feedback restraint. These components regulate repair programs as well as timekeeping. In Bmal1-deficient mice, epidermal healing was delayed through reduced epithelial proliferation and migration, altered ROMO1/ROS/SOD signaling, reduced SIRT1 activation, and impaired epithelial repair; topical antioxidant treatment partly rescued the phenotype [120]. Per2 deletion produced the opposite response, accelerating closure and increasing epithelial tongue formation, proliferation, angiogenic activity, myofibroblast presence, periostin organization, and collagen maturation [121]. These opposite phenotypes arise from different downstream nodes: Bmal1 acts through the ROMO1/ROS/SOD axis and epithelial proliferation, Per2 through myofibroblast appearance, periostin organization, and collagen maturation. Clock-directed therapy must therefore be specified to a component and its downstream pathway rather than framed as global enhancement or suppression of rhythm.

The most direct miRNA–clock link is the macrophage miR-155–BMAL1 axis: miR-155 targets Bmal1 and participates in circadian control of innate immunity [122]. This connects an inflammatory miRNA discussed above with a clock component capable of altering macrophage responsiveness. A broader interaction framework has been proposed across keratinocytes, macrophages, and fibroblasts [31]. Diabetes-associated metabolic and inflammatory disruption, including altered mitochondrial metabolic oscillations in type 2 diabetes [123], makes such coupling plausible. Nevertheless, time-resolved human studies and paired data on wound region, clock phase, cell type, and miRNA abundance remain lacking. Current evidence supports a possible interaction. A causal clock–miRNA axis in DFUs remains to be established.

Therapeutic studies support circadian-linked wound modulation, not chronomodulated miRNA therapy. A melatonin-loaded hydrogel improved repair under experimental circadian disruption by reducing oxidative stress and inflammation [124], and preclinical work implicates tenascin C and interleukin-10 in melatonin-associated fibroblast repair [125]. In a double-blind pilot trial involving 30 participants and 53 grade 1–2 DFUs, topical 1% melatonin hydrogel reduced ulcer number and grade and improved healing proportion over 60 days [126], although the between-group difference in ulcer-size reduction was not significant in a small sample carrying multiple ulcers per participant. This preclinical-to-clinical gap is itself informative: animal studies control the intensity and duration of circadian disruption, whereas participants differ in rhythm background, ulcer etiology, and concomitant medication. The route forward is stratification by rhythm phenotype, such as endogenous melatonin phase or sleep quality, combined with time-stamped sampling and head-to-head comparison of timing-aware and timing-agnostic delivery, rather than larger unstratified trials.

## 7. Toward Stage- and Time-Aware Therapeutic Design

The therapeutic implication of a temporal miRNA framework is not simple replacement or inhibition of a single dysregulated miRNA. Rather, DFU treatment design first requires definition of the dominant wound-state problem: persistent inflammation, delayed epithelial migration, insufficient angiogenesis, defective granulation tissue formation, abnormal matrix remodeling, or coexistence of several non-synchronized states within different wound regions. A small phase-linked miRNA panel may eventually support dynamic wound phenotyping, but time-resolved human DFU cohorts linking local miRNA profiles, wound region, cell state, treatment response, and durable closure are still lacking. Whether circulating miRNAs can report that local state is also unresolved: paired blood and wound-margin sampling has been performed in DFU patients [52] and plasma miR-126 correlated with vascular measures in a treatment model [73], but plasma, wound edge, and wound bed have not been resolved simultaneously and longitudinally in the same patients, so blood-based stage assignment cannot yet substitute for local sampling. A prospective study of 10 patients linked miRNA profiles in autologous platelet-rich-plasma-derived extracellular vesicles to wound closure during follow-up [127]. This finding concerns PRP-EV profiles during PRP treatment and does not establish miRNA-based staging of the wound itself. A stage-aware framework linking phase-linked biomarkers, delivery barriers, and spatiotemporally controlled delivery strategies is summarized in Figure 4 and Table 3. Because elapsed duration may not reliably indicate biological state in chronic DFUs (Section 2), Table 3 organizes the proposed interventions by biological stage rather than by fixed intervals after ulcer onset.

Table 3 distinguishes three levels of therapeutic evidence. Tier A denotes clinical efficacy in patients with DFUs. Tier B denotes human pharmacodynamic evidence, including the healthy-volunteer studies of MRG-110 and remlarsen [77,96]. Tier C denotes preclinical evidence, with the model and outcome stated in each row. The studies reviewed here do not establish Tier A evidence for a defined miRNA mimic or inhibitor. Clinical findings for an EV product support that product, but do not establish the efficacy of an individual miRNA cargo [111].

Because DFU repair involves multiple failed programs, stage-aware therapeutic design is unlikely to be achieved through single-miRNA correction alone. Huang et al. provided direct experimental support for this point by designing a miRNA cocktail composed of LNA-modified mimics of miR-19b-3p, miR-132-3p, and miR-31-5p together with a miR-92a-3p inhibitor [128]. This combination was selected to cover inflammation resolution, re-epithelialization, granulation tissue formation, and angiogenesis. In diabetic wound models, the cocktail attenuated inflammation while enhancing re-epithelialization, angiogenesis, granulation tissue formation, and nerve regeneration, and it showed stronger pro-healing effects than individual miRNA treatment [128]. The cocktail components were administered simultaneously. The results support combined miRNA treatment, but do not establish a benefit of sequential dosing at different healing stages.

The delivery system determines whether this stage logic can be implemented. Naked miRNA mimics or inhibitors are limited by instability, low tissue retention, inefficient cellular uptake, and off-target distribution. Biomaterial-supported extracellular vesicle platforms are therefore a practical bridge between molecular design and wound-bed delivery. Topical cerium-oxide-nanoparticle miR-146a delivery in a pluronic lecithin organogel has recently extended this principle to a needle-free formulation, enhancing diabetic wound healing in preclinical models [129]. Li et al. engineered miR-146a-loaded exosomes that were released from a silk fibroin patch; the system targeted IRAK1-related NF-κB signaling, reduced inflammatory cytokines, and promoted diabetic wound repair with improved granulation, re-epithelialization, collagen deposition, and neovascularization in db/db mice [60]. This example fits the inflammation-resolution window and illustrates how a pro-resolving miRNA can be combined with a local retention scaffold rather than delivered as an unprotected molecule.

Anti-angiogenic miRNA inhibition provides a more translation-oriented example. MRG-110, an LNA-modified inhibitor of miR-92a-3p, accelerated angiogenesis and wound healing in db/db mouse and normal pig wounds [88]. Published human evidence is pharmacodynamic rather than therapeutic: in healthy adults, systemic MRG-110 produced dose- and time-dependent inhibition of miR-92a-3p and derepressed targets including ITGA5 and CD93 [96]. A separate completed Phase 1 registry (NCT03603431) describes local intradermal MRG-110 after experimental excisional wounds in 42 healthy volunteers, with results not yet posted [97]. MRG-110 therefore shows that a wound-relevant miRNA therapeutic has entered human evaluation, with efficacy for wound healing and DFU still to be established.

Spatiotemporal control is required when an anti-miR signal must reach a defined wound compartment during a defined repair window. Lucas et al. developed a light-inducible antimiR-92a in which photolabile protecting groups prevented activity until local irradiation; in diabetic mice, light activation locally inhibited miR-92a, derepressed Itga5 and Sirt1, and improved wound repair by stimulating proliferation and angiogenesis [130]. The key contribution lies in demonstrating that miRNA inhibition can be externally triggered in a defined wound region and treatment window, building on the underlying anti-miR-92a biology. Other groups have extended this principle using electrostatically assembled wound dressings [131] and VCAM-1-targeted nanoparticle gels [132] to achieve endothelium-preferential anti-miR delivery in diabetic wounds. Together, these studies support a delivery design principle: temporal precision must be paired with spatial and cell-type control.

Human skin pharmacodynamic evidence also exists for remodeling-oriented miRNA therapy. Remlarsen, a miR-29b mimic, repressed collagen-related pharmacodynamic biomarkers and fibroplasia in a double-blinded, placebo-randomized, within-subject trial in healthy volunteers with incisional wounds [77], establishing miRNA mimic activity in human skin. A recently published randomized trial of Wharton’s jelly mesenchymal stem cell-derived exosomes reported improved DFU healing compared with standard care or vehicle [111], although this was a complex extracellular-vesicle product whose active cargo was not assigned to a defined miRNA and which lost participants between enrollment and analysis. It therefore supports the extracellular-vesicle platform rather than a defined miRNA.

Taken together, current evidence provides a basis for a realistic translational roadmap. The strongest current claim is that coordinated, locally retained, and cell-targeted RNA delivery can improve multiple repair processes in preclinical diabetic wound models, while selected miRNA therapeutics have achieved human skin or healthy-volunteer pharmacodynamic proof-of-mechanism. In this framework, stage-aware therapeutic design means delivering the appropriate miRNA program to the appropriate cell population, wound region, dose range, and repair phase, rather than applying a fixed miRNA target to all DFUs.

Computational and machine-learning approaches are beginning to support this stage-aware framework. In an integrated machine-learning analysis combining proteomic, transcriptomic, and miRNA data from diabetic mouse wounds at multiple time points and systemic serum proteomics from human DFU patients, Sumpio et al. used LASSO regression to identify miR-1b, miR-107-3p, MMP-2, and HGF as candidate sensors of healing state in mice, and Fractalkine and FGF-2 as systemic correlates in humans [133]. Such cross-omics, time-resolved classifiers will not replace mechanistic miRNA biology, but they may help identify which patients, at which moment of repair, are most likely to benefit from a stage-aware miRNA intervention.

## 8. Conclusions and Future Perspectives

Healing in diabetic foot ulcers depends on coordination among inflammatory resolution, epithelial repair, angiogenesis, and matrix remodeling. miRNAs help regulate these processes, but their effects depend on the cell type, wound region, and biological stage. This review organizes the available findings into three interpretive patterns of temporal dysregulation. These patterns provide a framework for understanding disrupted repair, while circadian regulation remains a possible additional influence with limited direct evidence in human DFUs.

The therapeutic implication is to match the miRNA intervention to the wound’s current repair needs. A preclinical miRNA cocktail improved diabetic wound repair by acting on several cellular programs [128]. Human studies of MRG-110 and remlarsen demonstrate pharmacodynamic activity outside DFUs [77,96]. These findings support further investigation, but clinical efficacy of a defined miRNA mimic or inhibitor in DFUs remains unestablished.

The main evidence gap is how miRNA activity changes over time across wound regions and cell populations. Repeated sampling combined with single-cell or spatial profiling could help resolve these relationships [10,13]. Computational models may help identify healing states, but they require prospective validation [133]. Therapeutic studies must also address RNA stability, local retention, cell-specific delivery, safety, and consistent carrier manufacture.

Future studies should specify biological wound stage, sampling site, source and target cells, dose, and circadian phase when measured. Outcomes should extend beyond short-term wound-area reduction to durable closure, recurrence, infection, and tissue quality and function. The practical goal is to determine which miRNA intervention benefits which wound state, and how to deliver it to the relevant cells at the appropriate time.

## Figures and Tables

**Figure 1:**
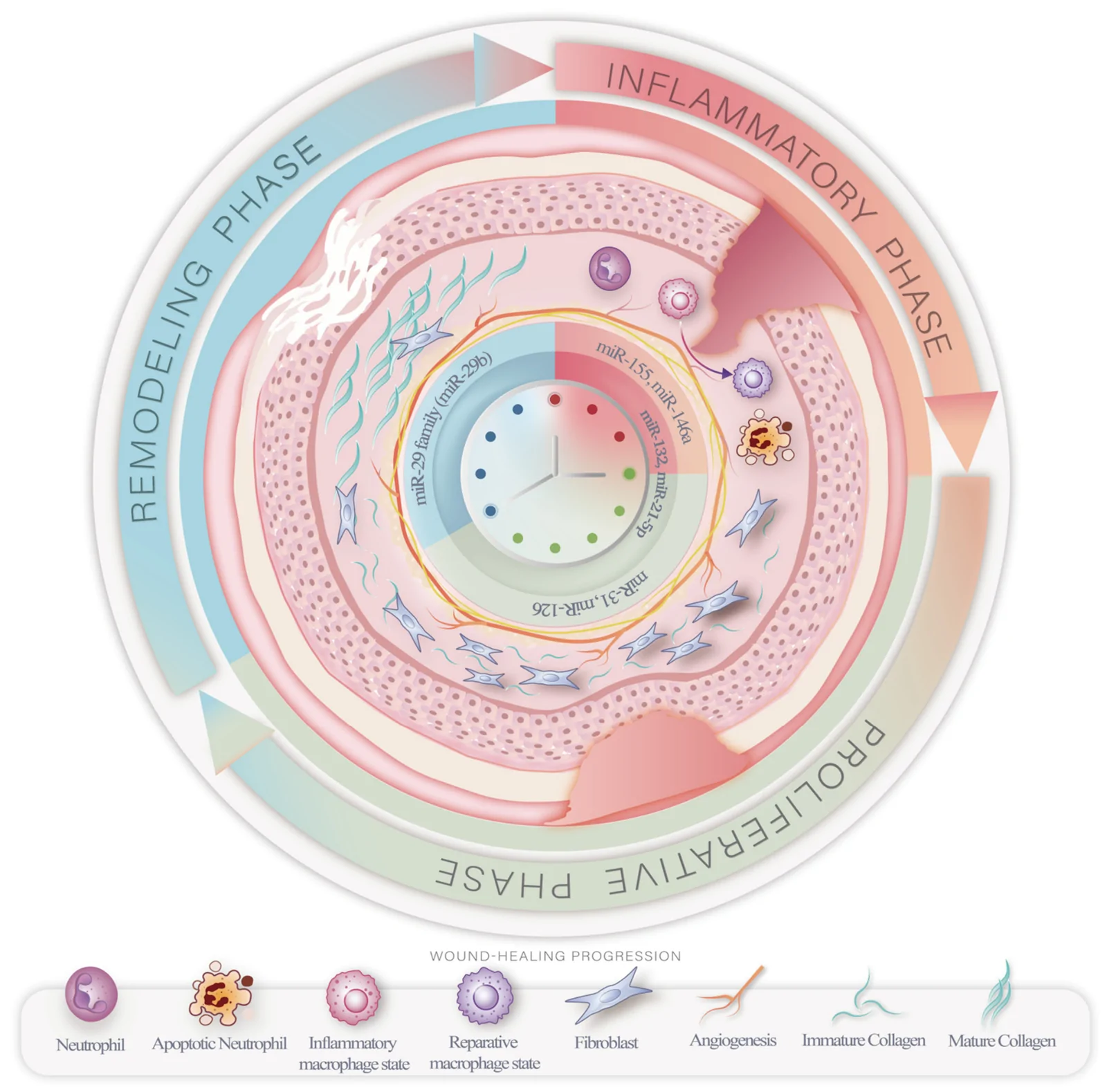
Wound-healing progression and representative miRNAs. The rings show overlapping repair phases, cellular events, and associated miRNAs. miR-21-5p denotes the strand-confirmed epithelial example [47,48], and miR-29b represents the miR-29 family. The central dial depicts healing progression, not a 24-h cycle. The outer arrows indicate progression through overlapping wound-healing phases. Pink, pale green, and light blue denote the inflammatory, proliferative, and remodeling phases, respectively.

**Figure 2:**
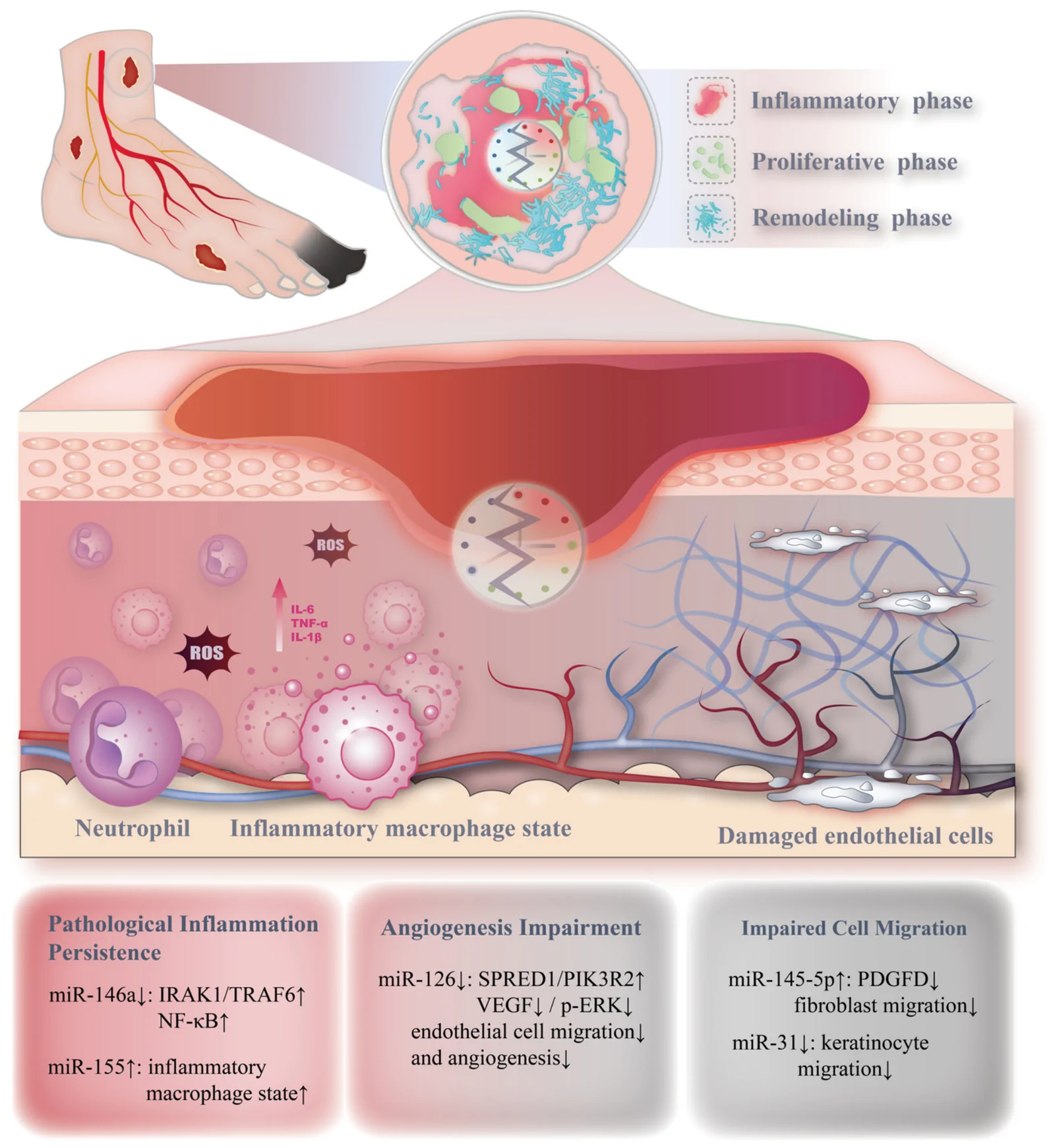
miRNA dysregulation and impaired repair in DFUs. The lower panels group examples by persistent inflammation, impaired angiogenesis, and reduced cell migration, not by temporal pattern. Elevated miR-145-5p with reduced PDGFD and reduced miR-31 illustrate impaired fibroblast and keratinocyte migration, respectively. Arrows indicate reported relative changes across models. Temporal-pattern assignments are summarized in Table 1. Upward and downward arrows indicate increases and decreases, respectively, in the cited experimental contexts. Pink, green, and blue in the upper-right legend denote the inflammatory, proliferative, and remodeling phases, respectively.

**Figure 3:**
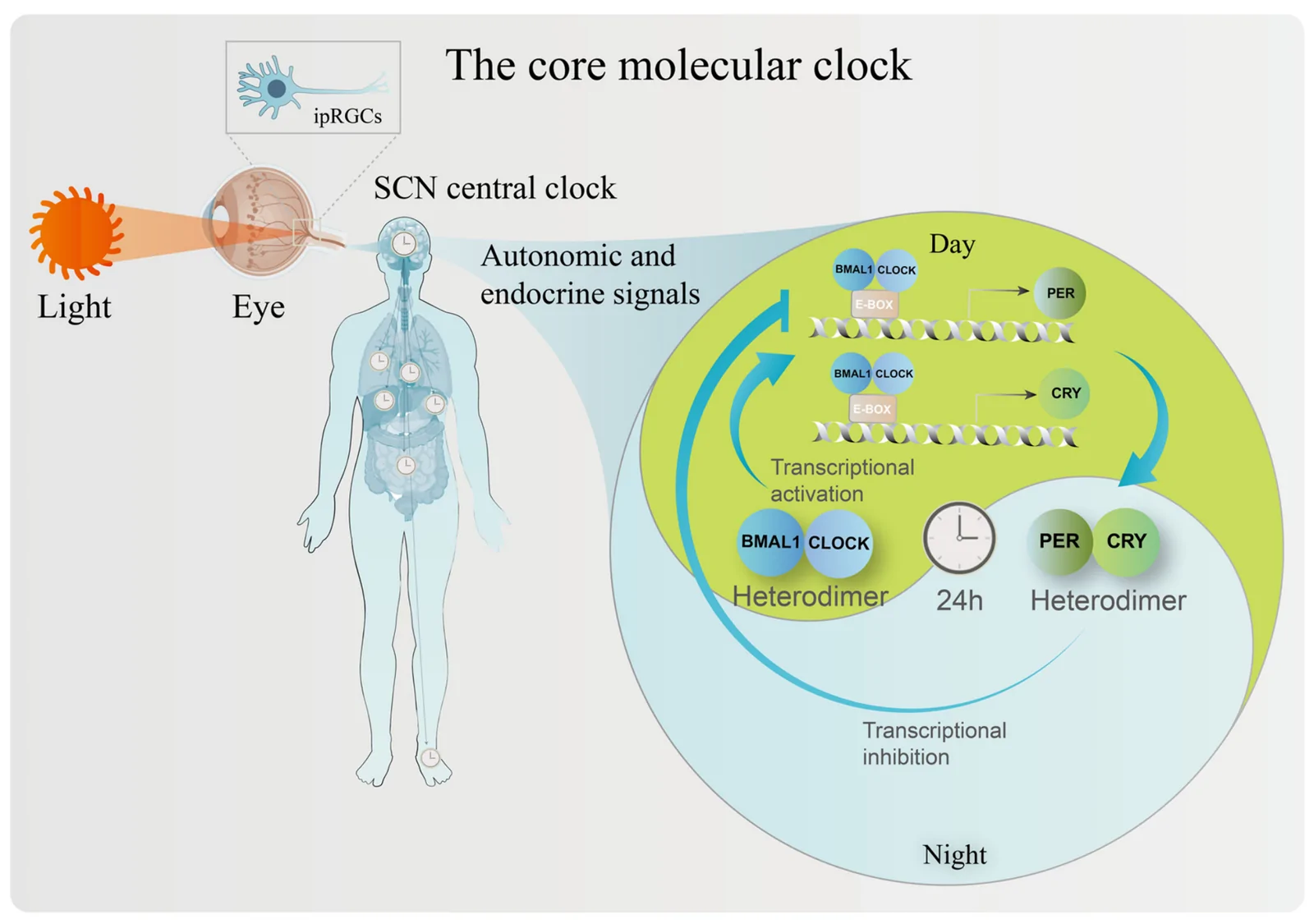
Circadian system and molecular clock. Light signals from intrinsically photosensitive retinal ganglion cells (ipRGCs) entrain the suprachiasmatic nucleus (SCN), which coordinates peripheral clocks. CLOCK–BMAL1 activates PER and CRY transcription, and PER–CRY complexes provide negative feedback. Day/night placement is schematic because clock timing varies across tissues and conditions. Arrows indicate signal flow and the molecular clock feedback loop. Green and light blue represent schematic day and night regions, respectively.

**Figure 4:**
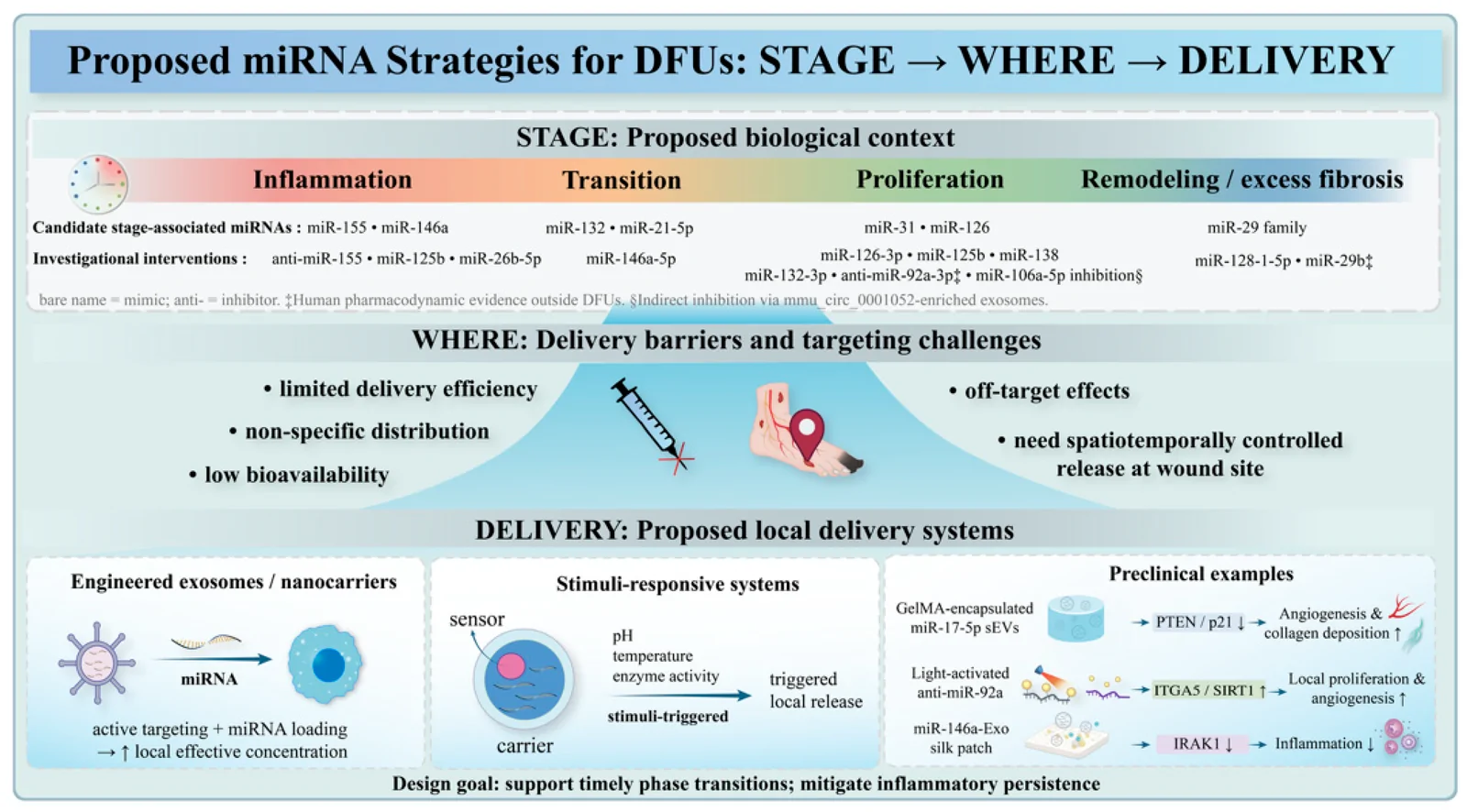
Proposed miRNA treatment design for DFUs. The upper panel links candidate stage-associated miRNAs and investigational interventions to biological repair contexts. The middle panel shows delivery barriers, and the lower panel shows local delivery approaches and preclinical examples. Stage markers and treatment timing remain investigational. ‡ Human pharmacodynamic evidence outside DFUs. § Indirect miR-106a-5p inhibition through circRNA-enriched EVs. Table 3 provides the evidence levels. ↑ and ↓ indicate increases and decreases, respectively.

**Table 1:** Three interpretive patterns of miRNA temporal dysregulation in DFUs: targets and pathological consequences.

miRNA	Time Point/Condition	Direction	Target	Pathway	Cell/Effect	Evidence/Model
**Pattern 1—Delayed or insufficient induction of reparative programs**						
miR-21-3p †	High glucose, 3, 24 h	Downward	SPRY1	RAS/MAPK (indirect)	↓ fibroblast proliferation, migration, ↓ collagen synthesis	In vitro fibroblasts [36].
miR-21-3p †	Days 4, 7, versus diabetic day 1	Downward	SPRY1	RAS/MAPK (indirect)	Reduced wound expression.	Mouse time-course, db/db/control. In vitro targets [36].
miR-146a	Days 1, 3, 7	Lower, no difference by day 14	IRAK1, TRAF6	TLR4/NF-κB	Increased inflammation, delayed closure.	Mouse time-course, db/db versus db/+ [37].
miR-126	Study days 0–15	Downward	—	—	Loss of vascular endothelial stability/integrity	Diabetic minipig time-course [38].
miR-210	Study days 0–15	Insufficient induction, inferred	ISCU, SDHD (reduced) [39]	—	Impaired metabolic adaptation to hypoxia	Minipig time-course [38]. Separate miR-210-3p intervention/in vitro [39].
**Pattern 2—Aberrant persistence of inflammatory/anti-reparative signals**						
miR-155	Study days 0–15	Higher than controls, declines over time	—	—	Sustained pro-inflammatory drive	Minipig time-course [38].
miR-122-5p	Mouse, day 14. Human, cross-sectional.	Upward	FN1, VEGF	NF-κB	↑ TNF-α, MMP9; ↓ fibroblast migration; ↓ FN1, α-SMA; delayed healing	Human cross-sectional, mouse comparison/intervention, in vitro [40].
miR-145-5p	High glucose, 15/25/35 mM (dose); 25 mM, 6–48 h (time)	Dose- and time-dependent increase	PDGFD	—	↓ fibroblast viability and migration; ↑ apoptosis	Human foreskin fibroblasts, in vitro [41].
miR-103	High glucose, 6, 12, 24 h	Upward	RCAN1	Calcineurin-NFAT (indirect)	↓ fibroblast proliferation, migration; ↑ apoptosis	Human dermal fibroblasts, in vitro [42].
miR-503	sEV exposure/intervention	Increased in pathological EVs	IGF1R	Macrophage sEV–miR-503/IGF1R	Impairs endothelial function and healing	Human cross-sectional, diabetic mouse intervention, in vitro [43].
miR-152-3p	High glucose, 3, 24 h	Upward	PTEN	PI3K/AKT, RAS/MAPK	↓ HUVEC proliferation and tube formation; ↑ apoptosis	In vitro HUVECs [44].
miR-152-3p	Days 4, 9	Upward	PTEN	PI3K/AKT, RAS/MAPK	Impaired endothelial repair	db/db mouse time-course, endothelial assays [44].
miR-26a	Days 1, 4, 9	Upward (sustained)	SMAD1, ID1, p27	BMP/SMAD1; VEGF	Altered endothelial function/angiogenesis timing	db/db mouse time-course, endothelial mechanisms [45].
**Pattern 3—Temporal inversion/phase-inappropriate expression**						
miR-21 (arm not specified)	Days 1, 3, 7, 14, 21	Higher, days 1, 14, 21, lower, days 3, 7	PTEN	PTEN/PI3K/NOX2/ROS	Inflammatory activation, phase-inappropriate expression.	Diabetic mouse time-course, macrophages in vitro [46].
miR-21 (arm not specified)	Study days 0–15	Lower than controls, no overall time effect	—	—	Reduced expression. Temporal inversion not demonstrated.	Minipig time-course [38].

Note: Patterns are inferred across models. Days follow injury, except minipig treatment-observation days [38]. Minipig controls received standard care without APG. Mouse/minipig comparisons use non-diabetic controls, with db/+ for [37]. Cell-culture hours are exposure durations. miR-210 expression [38] and intervention [39] are separate evidence. ↑ and ↓ indicate increases and decreases, respectively, relative to the comparator in the cited study. —, unreported. † [36] retains the source name despite a primer matching miR-21-5p and unavailable intervention sequences.

## Data Availability

No new data were created or analyzed in this study. Data sharing is not applicable to this article.

## Supplementary Materials

The following supporting information can be downloaded at: https://www.mdpi.com/article/10.3390/ijms27198491/s1. References [40,87,88,92,93,94,95,98,119,134,135,136,137,138,139,140,141,142,143,144,145,146,147,148,149,150] are cited in the supplementary materials.

## Abbreviations

The following abbreviations are used in this manuscript:

ADSC	Adipose-derived stem cell
AGE	Advanced glycation end product
Akt	Protein kinase B
ASK1	Apoptosis signal-regulating kinase 1
bFGF	Basic fibroblast growth factor
BMAL1	Brain and muscle ARNT-like protein 1
BMP	Bone morphogenetic protein
BMSC	Bone marrow mesenchymal stem cell
CCL2	C-C motif chemokine ligand 2
CD93	Cluster of differentiation 93
Cdc42	Cell division cycle 42
CLOCK	Circadian locomotor output cycles kaput
COL1A1	Collagen type I alpha 1 chain
COL3A1	Collagen type III alpha 1 chain
CRY	Cryptochrome
CTGF	Connective tissue growth factor
CXCL8	C-X-C motif chemokine ligand 8
db/db	Leptin receptor-deficient diabetic mouse
DFU	Diabetic foot ulcer
DLL4	Delta-like canonical Notch ligand 4
dpw	Days post-wounding
ECM	Extracellular matrix
EGFR	Epidermal growth factor receptor
EMP1	Epithelial membrane protein 1
ERK	Extracellular signal-regulated kinase
EV	Extracellular vesicle
FGF7	Fibroblast growth factor 7
FN1	Fibronectin 1
FoxO	Forkhead box O
GDF6	Growth differentiation factor 6
GelMA	Gelatin methacryloyl
GSTM5	Glutathione S-transferase mu 5
HB-EGF	Heparin-binding epidermal growth factor-like growth factor
HIF-1α	Hypoxia-inducible factor 1-alpha
HMGB1	High mobility group box 1
HSP47	Heat shock protein 47
HUVEC	Human umbilical vein endothelial cell
ID1	Inhibitor of DNA binding 1
IFN-γ	Interferon gamma
IGF1R	Insulin-like growth factor 1 receptor
IKKβ	Inhibitor of nuclear factor kappa-B kinase subunit beta
IL	Interleukin
ipRGC	Intrinsically photosensitive retinal ganglion cell
IRAK1	Interleukin-1 receptor-associated kinase 1
ISCU	Iron-sulfur cluster assembly enzyme
ITGA5	Integrin subunit alpha 5
JNK	C-Jun N-terminal kinase
LGR4	Leucine-rich repeat-containing G protein-coupled receptor 4
LNA	Locked nucleic acid
LPS	Lipopolysaccharide
MAPK	Mitogen-activated protein kinase
MIP-2	Macrophage inflammatory protein 2
miRNA	MicroRNA
MMP	Matrix metalloproteinase
MSC	Mesenchymal stem cell
NET	Neutrophil extracellular trap
NFAT	Nuclear factor of activated T cells
NF-κB	Nuclear factor kappa-light-chain-enhancer of activated B cells
NO	Nitric oxide
NOX2	NADPH oxidase 2
NPWT	Negative-pressure wound therapy
PDCD4	Programmed cell death protein 4
PDGF	Platelet-derived growth factor
PDGFD	Platelet-derived growth factor D
PER	Period
p-ERK	Phosphorylated extracellular signal-regulated kinase
PI3K	Phosphoinositide 3-kinase
PIK3R2	Phosphoinositide-3-kinase regulatory subunit 2
Pknox1	PBX/knotted 1 homeobox 1
PRP	Platelet-rich plasma
PTEN	Phosphatase and tensin homolog
PTP1B	Protein tyrosine phosphatase 1B
RAC1	Ras-related C3 botulinum toxin substrate 1
RAGE	Receptor for advanced glycation end products
RASA1	RAS p21 protein activator 1
RCAN1	Regulator of calcineurin 1
RHOB	Ras homolog family member B
ROMO1	Reactive oxygen species modulator 1
ROS	Reactive oxygen species
SCN	Suprachiasmatic nucleus
SDHD	Succinate dehydrogenase subunit D
sEV	Small extracellular vesicle
SHIP1	SH2 domain-containing inositol 5′-phosphatase 1
SHIP2	SH2 domain-containing inositol 5′-phosphatase 2
SIRT1	Sirtuin 1
SMAD	Mothers against decapentaplegic homolog
SNHG16	Small nucleolar RNA host gene 16
SOCS1	Suppressor of cytokine signaling 1
SOD	Superoxide dismutase
SPRED1	Sprouty-related EVH1 domain-containing protein 1
SPRY1	Sprouty RTK signaling antagonist 1
STAT3	Signal transducer and activator of transcription 3
STZ	Streptozotocin
TGF-β	Transforming growth factor beta
TIAM1	T-cell lymphoma invasion and metastasis 1
TIMP3	Tissue inhibitor of metalloproteinases 3
TLR	Toll-like receptor
TNF-α	Tumor necrosis factor alpha
VCAM-1	Vascular cell adhesion molecule 1
VEGF	Vascular endothelial growth factor
VEGFA	Vascular endothelial growth factor A
WT	Wild type
α-SMA	Alpha smooth muscle actin
