# Supplementary material for: Time to Heal: MicroRNA Temporal Dysregulation in Diabetic Foot Ulcers
Source: Int J Mol Sci. 2026 Sep 23;27(19):8491. doi: 10.3390/ijms27198491 (PMC13665434; doi:10.3390/ijms27198491)
Supplement: Supplementary file 1 [file ijms-27-08491-s001.zip › ijms-4528425-supplementary.pdf]

International Journal of
Molecular Sciences

MDPI

Time to Heal: MicroRNA Temporal Dysregulation in Diabetic Foot Ulcers

Lingyu Pan, Lingyun Zhao, Jinfeng Liu, You Mou, Kaili Wang, Xinyi Liu and Wei Hua

Supplementary Material

Table S1. Supporting miRNAs in temporal wound-healing regulation, organized by repair phase.

miRNA	Inflammatory stage	Proliferative stage	Remodeling stage	Key targets	Pathway	Evidence / model
miR-142 family	Migration, phagocytosis, bacterial clearance.	—	—	Rac1, Cdc42	Small GTPase–actin cytoskeleton	Infected non-diabetic mice, genetic intervention. Neutrophils, ex vivo/in vitro [134].
miR-223-3p / miR-223-5p	Neutrophil clearance, activation. 5p, M2 polarization.	3p and 5p delivery, improved closure in separate studies.	—	Pknox1, MAPK10, IRAK1 / RHOB	NF-κB/MAPK; macrophage polarization	Animal intervention/in vitro [135–137]. Review [138].
miR-34a-5p / miR-34c-5p	Elevated in venous ulcers. Proinflammatory in vitro.	34a-5p mimic delays mouse closure and epithelial repair.	—	LGR4	LGR4/GSK-3β/NF-κB	Human cross-sectional. Non-diabetic mouse intervention, keratinocytes in vitro [139].
miR-301a-5p	Reduced in early inflammation.	Increased by day 7. Delivery improves repair.	—	IKKβ	IKKβ/NF-κB	Mouse time-course/intervention, macrophages in vitro [140]
miR-23a/27a/24-2 cluster	Macrophage polarization.	Repair role inferred.	—	Polari-zation regulators	M1/M2 circuitry	Animal tumor models/in vitro, non-wound [141].
miR-125b	Context-dependent inflammation [S9].	ADSC EVs improve diabetic rat repair [119].	—	Direct targets uncon-firmed .	Reduced TLR4, IL-6, DLL4. Increased angiogenic markers.	In vitro [142]. Diabetic rat intervention, high-glucose HUVECs [119].
miR-497	NF-κB restraint.	Transition role inferred.	—	NF-κB regulators	NF-κB	Mechanistic review [143].
miR-23b	Reduced, days 1, 3, 5, recovers, 7, 14. Overexpression lowers cytokines.	Greater keratinocyte migration, α-SMA, collagen, contraction.	—	ASK1	ASK1/MAPK	Non-diabetic mouse time-course/intervention, keratinocytes in vitro [144].
miR-205 / miR-205-5p	—	SHIP2-linked migration. High-glucose effects vary.	—	SHIP2	PI3K-Akt; Rho-ROCK / cofilin	In vitro keratinocytes [145], separate high-glucose HaCaT [146].
miR-200b/c-3p	Reduced, day 2.	Day 10, 200b-3p recovers, 200c-3p remains low. Repression supports RAC1-dependent migration.	—	RAC1	TGF-β1–miR-200b/c-3p–RAC1	Non-diabetic mouse time-course/intervention, keratinocytes in vitro [147].
miR-210	Pattern 1, insufficient induction, inferred.	No overall time effect in diabetic wounds, rise in non-diabetic controls [40].	—	ISCU, SDHD [92]; PTP1B [148]	HIF hypoxic response; endothelial signaling	Minipig time-course [40]. Animal intervention/in vitro [92]. Non-wound PTP1B [148].
miR-195-5p / miR-205-5p	—	DFU wound-fluid EVs reduce VEGFA and angiogenesis.	—	VEGFA	VEGF angiogenic signaling	Human cross-sectional, DFU/control EVs. HUVECs in vitro, rat intervention [98].

miRNA	Inflammatory stage	Proliferative stage	Remodeling stage	Key targets	Pathway	Evidence / model
miR-199-5p	—	Endothelial EVs support Schwann-cell repair and nerve regeneration.	—	Repair-phenotype regulators	Exosome endothelial–neural crosstalk	Rat nerve intervention, Schwann cells in vitro, non-wound [149].
miR-182-5p	Inflammatory regulation.	Migration and fibroblast activity.	Collagen deposition / scar remodeling.	FoxO1, FoxO3	NF-κB, TGF-β, PI3K-Akt	Human scar/normal-wound samples, pairing unconfirmed, non-DFU-specific. Keratinocytes/fibroblasts in vitro [150].
miR-503	Dysregulated macrophage EVs.	Macrophage EVs impair endothelial repair.	—	IGF1R	Macrophage sEV–miR-503/IGF1R	Human cross-sectional, STZ-diabetic mouse intervention, macrophage/endothelial assays in vitro [87].
miR-152-3p	High glucose, increased, 3, 24 h.	Increased, days 4, 9. Inhibition improves endothelial repair.	—	PTEN	PTEN/PI3K-Akt, RAS/MAPK	Diabetic mouse time-course/intervention, HUVECs in vitro [88].
miR-145-5p	Glucose, 15/25/35 mM. 25 mM, 6–48 h.	Reduced fibroblast viability, migration. Increased apoptosis.	—	PDGFD	—	Human foreskin fibroblasts in vitro [93].
miR-103	High glucose, increased, 6, 12, 24 h.	Reduced fibroblast proliferation, migration. Increased apoptosis.	—	RCAN1	Calcineurin-NFAT (indirect)	Human dermal fibroblasts in vitro [94].
miR-26a	Increased, days 1, 4, 9.	Altered endothelial function and angiogenesis.	—	SMAD1, ID1, p27	BMP/SMAD1; VEGF	db/db mouse time-course/intervention, endothelial assays in vitro [95].

Note: dpw, days post-wounding. "—" means not reported or not stage-mapped. Citations follow the corresponding evidence or mechanism. Rows identify evidence sources, study designs, and secondary syntheses. Stage associations are functional interpretations, not validated human DFU trajectories. Cell-culture exposure times do not define wound stages. In Ref. [40], day 0 is the treatment-observation baseline, and controls received standard care without APG. Numerical citations refer to the main reference list, and S-prefixed citations to the supplementary list. Wound days are counted after injury, except treatment-observation days in Ref. [40]. EV, extracellular vesicle.

Supplementary References

134. Tanaka, K.; Kim, S.E.; Yano, H.; Matsumoto, G.; Ohuchida, R.; Ishikura, Y.; Araki, M.; Araki, K.; Park, S.; Komatsu, T.; et al. MiR-142 Is Required for Staphylococcus aureus Clearance at Skin Wound Sites via Small GTPase-Mediated Regulation of the Neutrophil Actin Cytoskeleton. *J. Investig. Dermatol.* **2017**, *137*, 931–940, https://doi.org/10.1016/j.jid.2016.11.018.
135. De Kerckhove, M.; Tanaka, K.; Umehara, T.; Okamoto, M.; Kanematsu, S.; Hayashi, H.; Yano, H.; Nishiura, S.; Tooyama, S.; Matsubayashi, Y.; et al. Targeting miR-223 in neutrophils enhances the clearance of Staphylococcus aureus in infected wounds. *EMBO Mol. Med.* **2018**, *10*, https://doi.org/10.15252/emmm.201809024.
136. Saleh, B.; Dhaliwal, H.K.; Portillo-Lara, R.; Shirzaei Sani, E.; Abdi, R.; Amiji, M.M.; Annabi, N. Local Immunomodulation Using an Adhesive Hydrogel Loaded with miRNA-Laden Nanoparticles Promotes Wound Healing. *Small* **2019**, *15*, e1902232, doi:10.1002/smll.201902232.
137. Liu, X.; Jin, S.; Liu, J.; Xu, X. MiR-223–3p overexpressed adipose mesenchymal stem cell-derived exosomes promote wound healing via targeting MAPK10. *Acta Histochem.* **2023**, *125*, 152102, https://doi.org/10.1016/j.acthis.2023.152102.
138. Jiao, P.; Wang, X.-P.; Luoreng, Z.-M.; Yang, J.; Jia, L.; Ma, Y.; Wei, D.-W. miR-223: An Effective Regulator of Immune Cell Differentiation and Inflammation. *Int. J. Biol. Sci.* **2021**, *17*, 2308–2322, https://doi.org/10.7150/ijbs.59876.
139. Wu, J.; Li, X.; Li, D.; Ren, X.; Li, Y.; Herter, E.K.; Qian, M.; Toma, M.-A.; Wintler, A.-M.; Sérézal, I.G.; et al. MicroRNA-34 Family Enhances Wound Inflammation by Targeting LGR4. *J. Investig. Dermatol.* **2020**, *140*, 465–476.e11, https://doi.org/10.1016/j.jid.2019.07.694.

140. Li, Y.; Fu, Z.; Deng, C.; Qian, J.; Wang, Z.; Lu, Y.; Zhang, S.; Liu, G.; Wen, J.; Chen, Y.; et al. miR-301a-5p regulated IKKβ/NF-κB axis and macrophage polarization to accelerate skin wound healing. *Int. J. Biol. Macromol.* **2025**, *311*, 143995, https://doi.org/10.1016/j.ijbiomac.2025.143995.
141. Ma, S.; Liu, M.; Xu, Z.; Li, Y.; Guo, H.; Ge, Y.; Liu, Y.; Zheng, D.; Shi, J. A double feedback loop mediated by microRNA-23a/27a/24-2 regulates M1*versus*M2 macrophage polarization and thus regulates cancer progression. *Oncotarget* **2015**, *7*, 13502–13519, https://doi.org/10.18632/oncotarget.6284.
142. Tili, E.; Michaille, J.-J.; Cimino, A.; Costinean, S.; Dumitru, C.D.; Adair, B.; Fabbri, M.; Alder, H.; Liu, C.G.; Calin, G.A.; et al. Modulation of miR-155 and miR-125b Levels following Lipopolysaccharide/TNF-α Stimulation and Their Possible Roles in Regulating the Response to Endotoxin Shock. *J. Immunol.* **2007**, *179*, 5082–5089, https://doi.org/10.4049/jimmunol.179.8.5082.
143. Herter, E.K.; Landén, N.X. Non-Coding RNAs: New Players in Skin Wound Healing. *Adv. Wound Care* **2017**, *6*, 93–107, https://doi.org/10.1089/wound.2016.0711.
144. Li, H.; Han, X.; Zuo, K.; Li, L.; Liu, J.; Yuan, X.; Shen, Y.; Shao, M.; Pang, D.; Chu, Y.; et al. miR-23b promotes cutaneous wound healing through inhibition of the inflammatory responses by targeting ASK1. *Acta Biochim. et Biophys. Sin.* **2018**, *50*, 1104–1113, https://doi.org/10.1093/abbs/gmy109.
145. Yu, J.; Peng, H.; Ruan, Q.; Fatima, A.; Getsios, S.; Lavker, R.M. MicroRNA-205 promotes keratinocyte migration *via* the lipid phosphatase SHIP2. *FASEB J.* **2010**, *24*, 3950–3959, https://doi.org/10.1096/fj.10-157404.
146. Zhang, L.; Hung, G.C.-C.; Meng, S.; Evans, R.; Xu, J. LncRNA MALAT1 Regulates Hyperglycemia Induced EMT in Keratinocyte via miR-205. *Non-Coding RNA* **2023**, *9*, 14, https://doi.org/10.3390/ncrna9010014.
147. Tang, H.; Wang, X.; Zhang, M.; Yan, Y.; Huang, S.; Ji, J.; Xu, J.; Zhang, Y.; Cai, Y.; Yang, B.; et al. MicroRNA-200b/c-3p regulate epithelial plasticity and inhibit cutaneous wound healing by modulating TGF-β-mediated RAC1 signaling. *Cell Death Dis.* **2020**, *11*, 1–17, https://doi.org/10.1038/s41419-020-03132-2.
148. 1Collado, A.; Jiao, T.; Kontidou, E.; Carvalho, L.R.R.A.; Chernogubova, E.; Yang, J.; Zaccagnini, G.; Zhao, A.; Tengbom, J.; Zheng, X.; et al. miR-210 as a therapeutic target in diabetes-associated endothelial dysfunction. *Br. J. Pharmacol.* **2024**, *182*, 417–431, https://doi.org/10.1111/bph.17329.
149. Huang, J.; Zhang, G.; Li, S.; Li, J.; Wang, W.; Xue, J.; Wang, Y.; Fang, M.; Zhou, N. Endothelial cell-derived exosomes boost and maintain repair-related phenotypes of Schwann cells via miR199-5p to promote nerve regeneration *J. Nanobiotechnology* **2023**, *21*, 1–25, https://doi.org/10.1186/s12951-023-01767-9,. erratum in J. Nanobiotechnology, 2024, 22, 81.
150. Amjadian, S.; Fatemi, M.J.; Moradi, S.; Hesaraki, M.; Mohammadi, P. mir-182-5p regulates all three phases of inflammation, proliferation, and remodeling during cutaneous wound healing. *Arch. Dermatol. Res.* **2024**, *316*, 1–12, https://doi.org/10.1007/s00403-024-03079-w.
